# Health-related quality of life in relation to symptomatic and radiographic definitions of knee osteoarthritis: data from Osteoarthritis Initiative (OAI) 4-year follow-up study

**DOI:** 10.1186/s12955-018-0979-7

**Published:** 2018-07-31

**Authors:** Soili Törmälehto, Mika E. Mononen, Emma Aarnio, Jari P. A. Arokoski, Rami K. Korhonen, Janne Martikainen

**Affiliations:** 1 Pharmacoeconomics and Outcomes Research Unit (PHORU), School of Pharmacy, University of Eastern Finland, Kuopio, Finland; 2 Department of Applied Physics, University of Eastern Finland, Kuopio, Finland; 3 Institute of Biomedicine, University of Turku, Turku, Finland; 4 Department of Physical and Rehabilitation Medicine, Helsinki University Hospital, Helsinki, Finland; 5 University of Helsinki, Helsinki, Finland; 6 Diagnostic Imaging Centre, Kuopio University Hospital, Kuopio, Finland

**Keywords:** Knee osteoarthritis, Health-related quality of life, SF-12, SF-6D

## Abstract

**Background:**

The purpose was to quantify the decrement in health utility (referred as disutility) associated with knee osteoarthritis (OA) and different symptomatic and radiographic uni- and bilateral definitions of knee OA in a repeated measures design of persons with knee OA or at increased risk of developing knee OA.

**Methods:**

Data were obtained from the Osteoarthritis Initiative database. SF-12 health-related quality of life was converted into SF-6D utilities, and were then handled as the health utility loss by subtracting 1.000 from the utility score, yielding a negative value (disutility). Symptomatic OA was defined by radiographic findings (Kellgren-Lawrence, K-L, grade ≥ 2) and frequent knee pain in the same knee. Radiographic OA was defined by five different definitions (K-L ≥ 2 unilaterally / bilaterally, or the highest / mean / combination of K-L grades of both knees). Repeated measures generalized estimating equation (GEE) models were used to investigate disutility in relation to these different definitions.

**Results:**

Utility decreased with worsening of symptomatic or radiographic status of knee OA. The participants with bilateral and unilateral symptomatic knee OA had 0.03 (*p* < 0.001) and 0.02 (*p* < 0.001) points lower utility scores, respectively, compared with the reference group. The radiographic K-L grade 4 defined as the mean or the highest grade of both knees was related to a decrease of 0.04 (*p* < 0.001) and 0.03 (*p* < 0.001) points in utility scores, respectively, compared to the reference group.

**Conclusions:**

Knee OA is associated with diminished health-related quality of life. Health utility can be quantified in relation to both symptomatic and radiographic uni- and bilateral definitions of knee OA, and these definitions are associated with differing disutilities. The performance of symptomatic definition was better, indicating that pain experience is an important factor in knee OA related quality of life.

**Electronic supplementary material:**

The online version of this article (10.1186/s12955-018-0979-7) contains supplementary material, which is available to authorized users.

## Background

Osteoarthritis (OA) is a chronic and degenerative joint disease. It is the most common type of arthritis, and it affects most frequently hips, knees, and hands [1]. Knee OA is a major cause of disability and loss of function among older adults, and it causes a major burden both to individuals and health care systems [2, 3]. Older age and obesity are significant risk factors of knee OA [4, 5], and with the continuously aging population and increasing prevalence of obesity, the burden of knee OA is anticipated to increase.

While knee OA manifests pain, stiffness, and daily activity deficits, it causes deterioration in patient-reported health-related quality of life (HRQoL). OA is associated with strong negative effect on HRQoL [6, 7], and bilateral knee pain, other joint pain comorbidity, and inadequate pain relief in conjunction with knee OA have been shown to be associated with even poorer quality of life [8–10]. Correspondingly, total knee replacement has been reported to improve patients’ quality of life [11].

In chronic conditions such as knee OA, HRQoL is one of the most commonly used patient-reported outcome metrics. In knee OA research literature, several disease specific instruments have been used under the label of HRQoL. The Western Ontario and McMaster Universities Osteoarthritis Index (WOMAC) [12] and The Knee injury and Osteoarthritis Outcome Score (KOOS) [13], and their subscales, are widely used questionnaires to assess patient-reported outcomes related to knee OA in clinical trials. WOMAC questionnaire assesses pain, stiffness, and functional limitation [12], while KOOS includes separate subscales for pain, symptoms, function in daily living, function in sport and recreation, and knee-related quality of life [13]. The benefit of these disease-spesific instruments is that they cover dimensions relevant to knee OA [14]. However, disease-spesific HRQoL measures do not provide preference-based utility values needed in health economic analyses. Instead, single, general, and calibrated preference-based health utility scores are needed to incorporate the quantity of life (years) and quality of life into quality-adjusted life years (QALYs) [15]. QALY is commonly used as an outcome measure in health economic analyses and health care decision-making.

In health economic analyses, information on how health utility values response to the disease-spesific health states is essential. However, in the case of knee OA, the gold standard of knee OA definition is currently unavailable, and variety of criteria and definitions have been used in previous research studies [16]. Previously, EQ-5D (EuroQol-5 dimensions questionnaire) health utility values in relation to different knee OA definitions have been assessed in two separate studies [17, 18]. Both of the studies used an unilateral knee OA definition. Consequently, the information on health utility in relation to different definitions of knee OA is scarce, and particularly information on health utility in relation to bilateral definition of knee OA is lacking. Therefore, the objective of the present study was to quantify the preference-based health utility values associated with different symptomatic and radiographic uni- and bilateral definitions of knee OA in a repeated measures design of persons with symptomatic knee OA or at increased risk of developing knee OA.

## Methods

### Data sources

Data used in the preparation of this article were obtained from the open access Osteoarthritis Initiative (OAI) study (http://www.oai.ucsf.edu/), which is a multi-center, longitudinal cohort study on knee OA [19]. The study incorporates a progression, an incidence, and a reference sub-cohort. Subjects in the progression sub-cohort have symptomatic knee OA at baseline, while subjects in the incidence sub-cohort are at increased risk of developing it. Reference sub-cohort subjects have neither symptomatic knee OA nor eligibility risk factors at baseline. The specific eligibility risk factors and ethical issues are described in detail in *Osteoarthritis Initiative Study Protocol* [19]. Ethical approval for collecting all subject information was provided by the OAI. Informed consent was obtained from all individual participants included in the study. Applied OAI datasets are listed in additional file (see Additional file 1). Data from baseline and follow-up visits at 12, 24, 36 and 48 months were applied in the study.

### Preference-based health utility index

A mapping algorithm (https://www.sheffield.ac.uk/) was used to convert 12-item Short Form Health Survey (SF-12) data from the OAI study into SF-6D utility scores [20]. SF-12 is a measure of general health covering eight health domains. The SF-6D estimates a preference-based single index utility measure from SF-12 using general population values and standard gamble valuation technique. SF-6D scores fall on the scale from 0 (death) to 1 (perfect health). The worst SF-6D score, excluding death, is 0.291 (‘floor effect’). We handled SF-6D utilities as decrement in health utility by subtracting 1.000 from the SF-6D utility score, yielding a negative value (referred as disutility).

### Definitions of knee OA

Kellgren-Lawrence (K-L) radiographic system classifies knee OA into five grades based on the severity of radiographic findings of joint space narrowing, osteophytes, sclerosis, and bone deformity [21]. K-L grade 0 indicates intact joint without any features of OA, and K-L grade 2 is considered the cut-off point of definite OA, while K-L grade 4 indicates severe OA. However, persons with K-L grade ≥ 2 may be asymptomatic and vice versa. Therefore, symptomatic definition of knee OA is considered more relevant. In prevalence studies, symptomatic knee OA is usually defined as the concurrent presence of radiographic findings (usually K-L grade ≥ 2) and frequent knee pain in the same knee [1, 22, 23].

In order to estimate the effect of different definitions on the HRQoL associated with knee OA, seven definitions of knee OA were studied. Two definitions were based on the symptomatic definition of knee OA (K-L grade ≥ 2 and knee pain, aching or stiffness on more than half the days during past month in the same knee): (a) 2-scale: no/yes (OA present either uni- or bilaterally), or (b) 3-scale: no/unilateral/bilateral knee OA. The remaining five definitions were based solely on the radiographic definition of knee OA: (c) 2-scale: no/yes (K-L grade ≥ 2 either uni- or bilaterally), (d) 3-scale: no/unilateral/bilateral K-L grade ≥ 2, (e) 5-scale unilateral definition: the highest K-L grade of both knees, (f) 9-scale bilateral definition: mean K-L grade of both knees, or (g) 15-scale bilateral definition: combination of K-L grades of both knees ((0;0), (1;0), (1;1) … (4;2), (4;3), (4;4)). Missing data regarding K-L grades was not imputed.

### Covariates

We used 11 covariates as adjusting variables. Demographics (age, gender, education, living status), clinical status (injuries, surgical history, body mass index (BMI), comorbid conditions, smoking status), and physical activity were selected as a standard set of adjusting variables according to Rolfson and co-authors [24]. We also included race into adjusting variables because of its association with pain level and health status [25, 26].

Injury status and surgical history were both dichotomized and based on the OAI query on ever (baseline) or since last visit (follow-up) injuring either knee badly enough to limit ability to walk for at least 2 days, and ever having knee surgery or arthroscopy (answers regarding left and right knee pooled). Self-reported race was dichotomized (White or Caucasian/Other than White) because the frequency in original groups ‘Black or African American’, ‘Asian’ and ‘other Non-White’ was low.

Age, living status, BMI, physical activity and comorbid conditions were categorized because the distribution of the covariates was skewed and in order to allow for nonlinear associations. Age was categorized into three groups (45–54, 55–64, and 65–79 years). Education based on the OAI query refers to the highest grade of school completed, and had three categories: primary/none (less than college), secondary (college graduate or some graduate school), and tertiary level (graduate degree).

Living status based on the OAI query was dichotomized as living alone or living with someone else. BMI based on physical examination was categorized into four groups (< 25, 25 to < 30, 30 to < 35, ≥35 kg/m2). Physical activity was based on the Physical Activity Scale for the Elderly (PASE) score, where higher scores indicate greater physical activity [27], and was divided into quintiles (0–90, 91–134, 135–175, 176–237, 238–526). Smoking status was categorized into three groups (never/former/current smoker) based on OAI questions on smoking pipe, cigars or cigarillos and smoking cigarettes. Comorbid conditions based on Charlson comorbidity index score [28] was dichotomized (none/> 0 comorbid conditions).

OAI data covered living status at baseline and year 3 follow-up visit, comorbid conditions at baseline, year 2 and 4 follow-up visits, and smoking status at baseline and year 4 follow-up visit. Missing data regarding living status, comorbid conditions and smoking status were imputed with the information available from the baseline visit or with the previous follow-up visit (i.e., last observation carried forward).

### Statistical methods

Descriptive statistics were used to summarize characteristics of OAI study participants. Repeated measures generalized estimating equation (GEE) models [29] were used to evaluate the population average [30] disutility in relation to the symptomatic and radiographic definitions of knee OA in the longitudinal data in order to take into account the repeated measurements of the same individual as observations of a same study subject are correlated. GEE modeling was used because it allows the inclusion of all baseline participants in the analyses rather than only those participants who remained in the study and had data available at all follow-up visits. By utilizing a GEE model, we were able to include data for participants who dropped out of the study, or who did not have radiographic knee examination in all visits. All GEE-models were specified using a normal (Gaussian) distribution, an identity link function and an unstructured correlation matrix. The assumption of normal distribution is justified by the large number of observations, and because by that means we avoided transformation of explanatory variable values equal to zero or one, which is required for example in gamma and beta regressions. The inspection of disutility distributions is presented in the additional file (see Additional file 2). The repeated within-subject time-variable was the visit number (0, 1, 2, 3 or 4). All GEE models were adjusted for the 11 covariates mentioned earlier. The dependent variable was the SF-6D disutility score and the independent variable was a symptomatic or radiographic definition of knee OA. Quasi Likelihood under Independence Model Criterion was used to study models’ goodness of fit. Estimated marginal means and 95% Wald confidence intervals of disutility scores were compared between different subgroups of knee OA definitions. Participants with no knee OA on the basis of different definitions were regarded as the reference group. Results are presented also as minimally important difference (MID) values using a score difference of ≥0.027 as a cut-point for MID [31]. In all statistical analyses, conducted with IBM SPSS for Windows, version 23.0, the level of statistical significance was considered as *p* < 0.05.

## Results

### Population characteristics

**Fig. 1:**
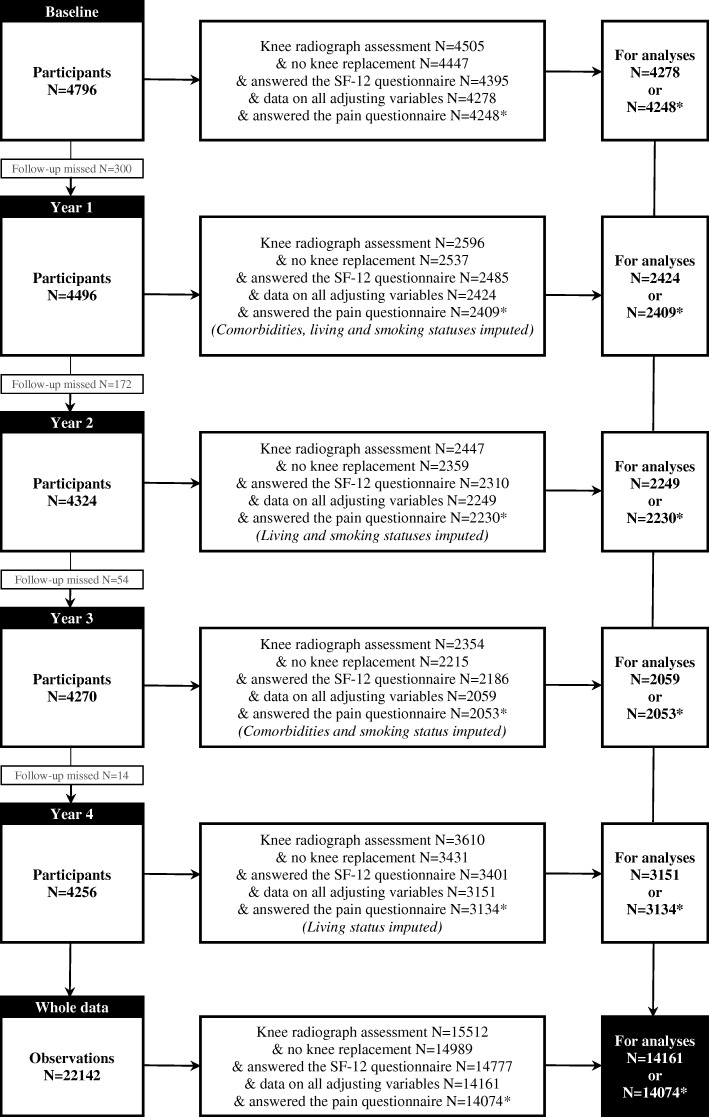
The flow diagram of the OAI study participants and number of observations. * Pain questionnaire required for the definition of symptomatic knee OA (K-L grade ≥ 2 and knee pain on more than half the days during past month in the same knee)

The flowchart of the cohort definition and number of observations eligible for the adjusted GEE analyses is presented in Fig. 1. In order to be eligible for analyses, participants had to have knee radiograph assessment available for both knees and to have answered the SF-12 questionnaire in full at the same study visit at least once. In addition, participants with partial or total knee replacement (KR) were excluded at baseline. Altogether, 4278 participants were eligible at baseline and had data on all adjusting variables. Participants having a partial or total KR at least for one knee during the follow-up were censored once after the replacement. There were 14,161 observations eligible for analyses according to the radiographic definition of knee OA during the 4-year follow-up. In order to be eligible for analyses according to the symptomatic definition of knee OA, participants had to have answered the knee pain query also (14,074 observations eligible for analyses). The main reason for missing data in the present analyses was the missing data on K-L grades for 42, 43 and 44% of participants at study visits 1, 2 and 3, respectively (see Fig. 1 and Additional file 3). Fifty-three percent of participants eligible for the analyses had three or more observations (see Additional file 3).

The baseline characteristics of participants are presented in Table 1. At baseline, the mean age was 61.1 years (standard deviation (SD) 9.2), and 58% of participants were female. The mean BMI was 28.5 (SD 4.8), and 76% of participants had no comorbid conditions at baseline. Fifty-seven and 78% of participants reported no knee injuries and no knee surgical history, respectively, at baseline. The characteristics of participants during follow-up are presented in additional file (see Additional file 3).

**Table 1:** Baseline population characteristics of participants eligible for data analyses

Variable	N (%)	Mean (SD)
Participants	4278 (100.0)	
Age, years		61.1 (9.2)
45–54	1264 (29.5)	
55–64	1393 (32.6)	
> 65	1621 (37.9)	
Gender		
Male	1782 (41.7)	
Female	2496 (58.3)	
Race		
White or Caucasian	3458 (80.8)	
Other	820 (19.2)	
Education^a^		
Tertiary	1336 (31.2)	
Secondary	1277 (29.9)	
None / Primary	1665 (38.9)	
Living status (number of persons)		1.3 (1.1)
Live alone	928 (21.7)	
Living with someone else	3350 (78.3)	
BMI, kg/m2		28.5 (4.8)
< 25	1040 (24.3)	
25 to < 30	1690 (39.5)	
30 to < 35	1122 (26.2)	
≥35	426 (10.0)	
Comorbidities		0.4 (0.8)
score 0	3254 (76.1)	
score > 0	1024 (23.9)	
Physical activity, PASE^b^		162.1 (81.8)
238–580	798 (18.7)	
176–237	853 (19.9)	
135–175	866 (20.2)	
91–134	871 (20.4)	
0–90	890 (20.8)	
Smoking status		
Never	1977 (46.2)	
Former	1908 (44.6)	
Current	393 (9.2)	
Knee injuries		
No	2424 (56.7)	
Yes	1854 (43.3)	
Knee surgical history		
No	3341 (78.1)	
Yes	937 (21.9)	
Disutility (SF-6D) score		−0.199 (0.120)

*Abbreviations*: *BMI* body mass index; Comorbidites Charlson comorbidity index score, *PASE* physical activity scale for the elderly, *SD* standard deviation

^a^The highest grade of school completed: tertiary (graduate degree), secondary (college graduate or some graduate school), and primary/none level (less than college)

^b^Physical activity PASE score quintiles (higher scores indicate greater physical activity)

### Knee OA according to different definitions

The prevalence of knee OA according to different definitions among participants at baseline and among all observations are presented in Table 2. At baseline, 57% of participants had K-L grade ≥ 2 in at least one knee, and 26% had knee OA according to the symptomatic definition of knee OA. At baseline, when using the definition of the highest K-L grade for knee OA, the most prevalent grades were 2 (30%) and 0 (28%), while the most prevalent mean K-L grades were 0.0 (28%) and 2.0 (17%). The most prevalent K-L grade combinations at baseline were (0;0) and (2;2) accounting for 28 and 13% of participants, respectively. The prevalence of knee OA according to different definitions during follow-up are presented in additional file (see Additional file 3).

**Table 2:** Prevalence of knee OA according to different definitions

Variable	Baseline N (%)	All observations N (%)
N	4278	14,161
Symptomatic OA status^a^ (2-scale)		
No	3134 (73.8)	9538 (67.8)
Yes, uni- or bilateral	1114 (26.2)	4536 (32.2)
Symptomatic OA status^a^ (3-scale)		
No	3134 (73.8)	9538 (67.8)
Yes, unilateral	762 (17.9)	2999 (21.3)
Yes, bilateral	352 (8.3)	1537 (10.9)
K-L grade ≥ 2 (2-scale)		
No	1860 (43.5)	3395 (24.0)
Yes, uni- or bilateral	2418 (56.5)	10,766 (76.0)
K-L grade ≥ 2 (3-scale)		
No	1860 (43.5)	3395 (24.0)
Yes, unilateral	1112 (26.0)	4785 (33.8)
Yes, bilateral	1306 (30.5)	5981 (42.2)
The highest K-L grade		
0	1199 (28.0)	2051 (14.5)
1	661 (15.5)	1344 (9.5)
2	1288 (30.1)	5544 (39.1)
3	841 (19.7)	3693 (26.1)
4	289 (6.8)	1529 (10.8)
Mean of K-L grades		
0.0	1199 (28.0)	2051 (14.5)
0.5	383 (9.0)	774 (5.5)
1.0	612 (14.3)	2032 (14.3)
1.5	550 (12.9)	2346 (16.6)
2.0	728 (17.0)	3151 (22.3)
2.5	391 (9.1)	1660 (11.7)
3.0	313 (7.3)	1470 (10.4)
3.5	100 (2.3)	566 (4.0)
4.0	2 (0.0)	111 (0.8)
Combination of K-L grades		
(0;0)	1199 (28.0)	2051 (14.5)
(1;0)	383 (9.0)	774 (5.5)
(1;1)	278 (6.5)	570 (4.0)
(2;0)	334 (7.8)	1462 (10.3)
(2;1)	403 (9.4)	1714 (12.1)
(2;2)	551 (12.9)	2368 (16.7)
(3;0)	147 (3.4)	632 (4.5)
(3;1)	123 (2.9)	525 (3.7)
(3;2)	340 (7.9)	1466 (10.4)
(3;3)	231 (5.4)	1070 (7.6)
(4;0)	54 (1.3)	258 (1.8)
(4;1)	51 (1.2)	194 (1.4)
(4;2)	82 (1.9)	400 (2.8)
(4;3)	100 (2.3)	566 (4.0)
(4;4)	2 (0.0)	111 (0.8)

^a^K-L grade ≥ 2 and knee pain on more than half the days during past month in the same knee

### SF-6D disutility scores in relation to definitions of knee osteoarthritis

**Fig. 2:**
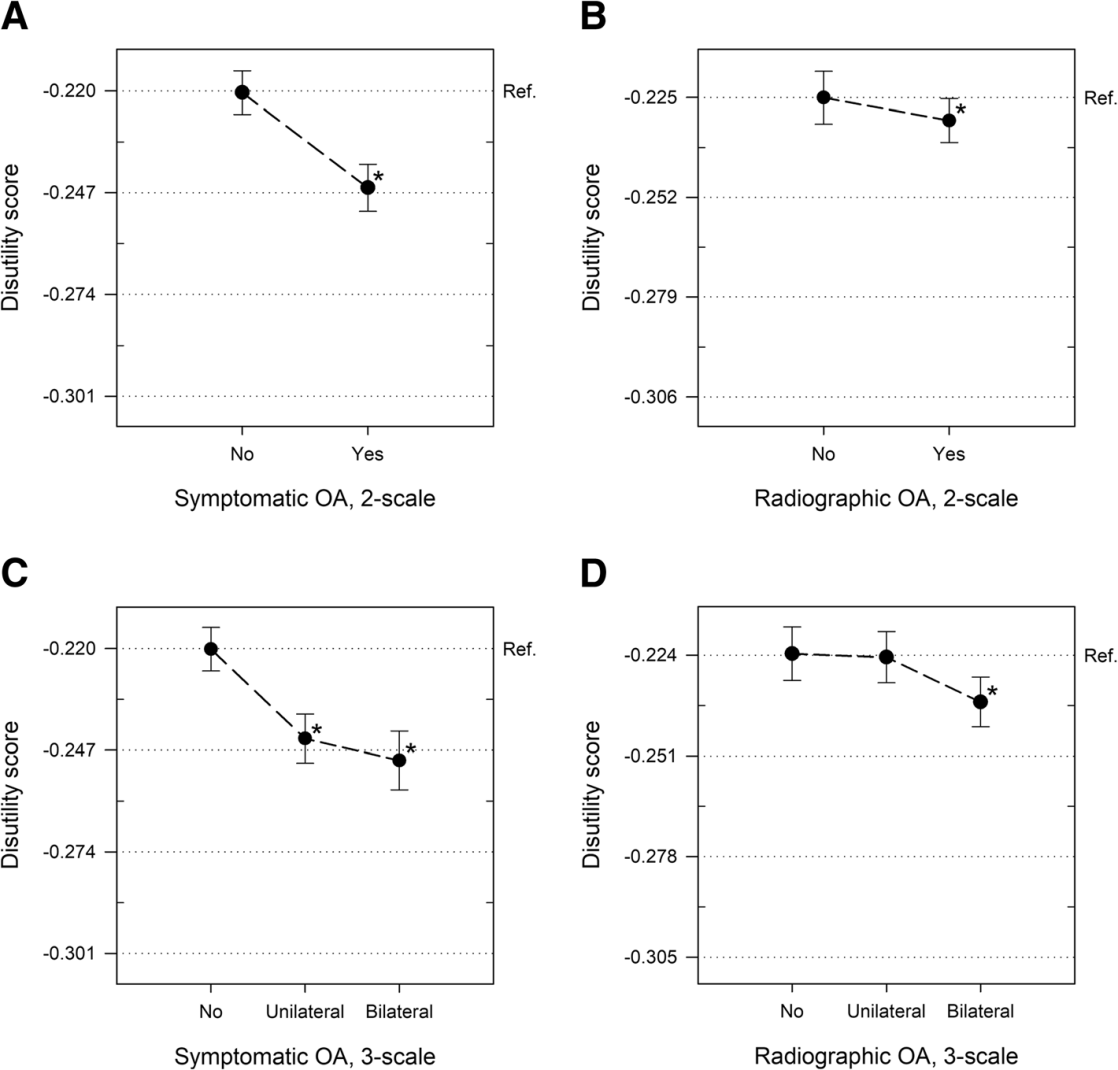
SF-6D disutility scores in relation to symptomatic and radiographic knee OA definitions. **a** 2-scale symptomatic knee OA (K-L grade ≥ 2 and frequent knee pain in the same knee in at least one knee) **b** 2-scale radiographic knee OA (K-L grade ≥ 2 in at least one knee) **c** 3-scale symptomatic knee OA (K-L grade ≥ 2 and frequent knee pain in the same knee) **d** 3-scale radiographic knee OA (K-L grade ≥ 2). Disutility of the best health state possible (no OA) is set as the horizontal reference line (dotted). Other horizontal lines (dotted) are set in 0.027 point intervals representing 1.0 MID (minimally important difference). Error bars are 95% confidence intervals. The mean difference is significant at the 0.05 level (*) in comparison to the best health state (no OA)

**Fig. 3:**
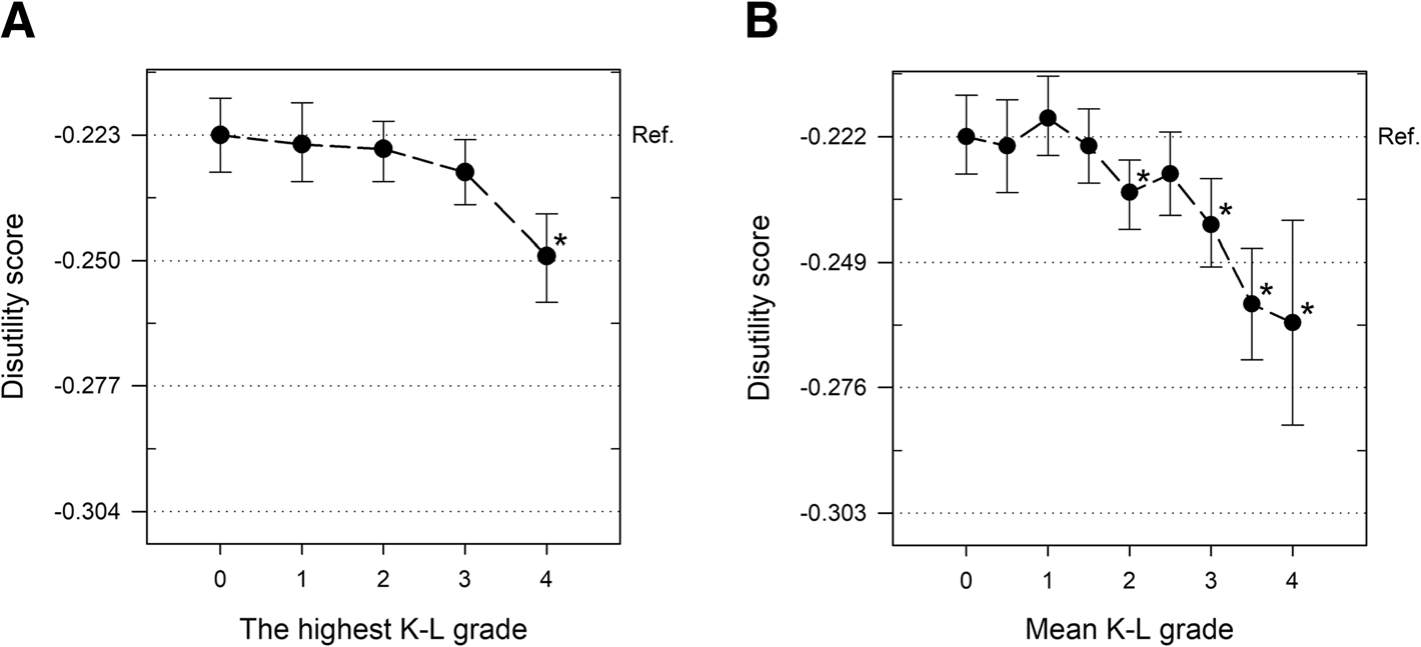
SF-6D disutility scores in relation to 5- and 9-scale radiographic knee OA definitions. **a** 5-scale radiographic knee OA (the highest K-L grade in both knees) or **b** 9-scale radiographic knee OA (mean of K-L grades in both knees). Disutility of the best health state possible (K-L grade 0) is set as the horizontal reference line (dotted). Other horizontal lines (dotted) are set in 0.027 point intervals representing 1.0 MID (minimally important difference). Error bars are 95% confidence intervals. The mean difference is significant at the 0.05 level (*) in comparison to the best health state (K-L grade 0)

**Fig. 4:**
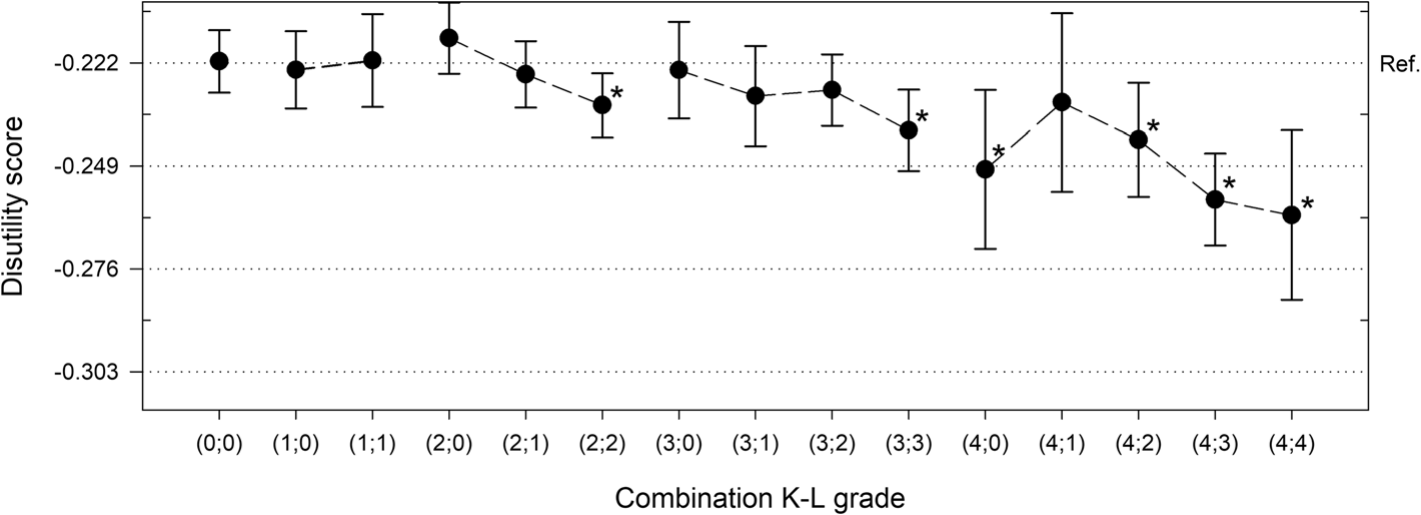
SF-6D disutility scores in relation to 15-scale radiographic knee OA definition. 15-scale radiographic knee OA (combination of K-L grades in both knees). Disutility of the best health state possible (K-L grade 0;0) is set as the horizontal reference line (dotted). Other horizontal lines (dotted) are set in 0.027 point intervals representing 1.0 MID (minimally important difference). Error bars are 95% confidence intervals. The mean difference is significant at the 0.05 level (*) in comparison to the best health state (K-L grade 0;0)

At baseline, the mean (SD) unadjusted SF-6D utility score of all study participants was 0.801 (0.120) indicating utility loss (disutility) equal to − 0.199 (see Table 1). The mean adjusted disutility scores in relation to different symptomatic and radiological definitions of knee OA are presented in Figs. 2, 3 and 4 and in more detail in additional file (see Additional file 4). The parameter estimates from GEE analyses are presented in additional file (see Additional file 4). Pairwise comparisons are presented in additional file (see Additional file 5).

The estimated disutility score increased with worsening of symptomatic or radiographic status of knee OA. Symptomatic and radiographic knee OA in at least one knee was associated with an increase of 0.026 and 0.006 (corresponding to 1.0 MID and 0.2 MID), respectively, in disutility scores compared with the reference group (Fig. 2a and b). Bilateral or unilateral symptomatic knee OA was associated with 0.030 (1.1 MID) or 0.024 (0.9 MID) higher disutility scores, respectively, compared with the reference group (Fig. 2c). The estimated increase in disutility was 0.013 points (0.5 MID) with bilateral radiographic knee OA compared with the reference group (Fig. 2d).

The estimated disutility scores increased by 0.027 and 0.040 points (1.0 MID and 1.5 MID) from the best to the worst among the highest and mean of K-L grades (Fig. 3a and b), respectively. Compared to a mean K-L grade value of 0.0, the estimated increase in disutility score was statistically significant with mean values of 2.0, 3.0, 3.5, and 4.0 (Fig. 3b). An additional file shows this in more detail (see Additional file 5).

Increase in estimated disutility scores from the best combination of K-L grades (0;0) to the worst (4;4) was the same (0.040 points, corresponding to 1.5 MID) as with mean K-L grade classification due to arithmetical reasons (Fig. 4). The disutility scores in health states (2;2), (3;3), (4;0), (4;2), (4;3), and (4;4) differed significantly from the reference health state (0;0) (Fig. 4). An additional file shows this in more detail (see Additional file 5).

The difference in disutility scores between the worst and best health state according to different definitions of knee OA was the greatest when using combinations or mean values of K-L grades. The differences in the remaining definitions can be ranked as follows: symptomatic OA (3-scale) > highest K-L grade > symptomatic OA (2-scale) > radiographic OA (3-scale) > radiographic OA (2-scale).

## Discussion

In the present study, we estimated health disutilities associated with knee OA and its different symptomatic and radiographic uni- and bilateral definitions. Our results show that the emergence of symptomatic knee OA and increasing radiographic K-L grade are in relation to statistically and clinically significant worsening of health utility measured by the SF-6D instrument. The worsening was particulary strong when the subject had symptomatic bilateral knee OA or a high mean value of K-L grades. Health disutility in bilateral knee OA differed from disutility associated with unilateral knee OA when using the radiographic definition. Based on our results, pain experience is an important factor in OA-related quality of life as the disutility score associated with knee OA was greater when taking pain experience into account (i.e., symptomatic definition) than when using only radiographic definition (K-L grade ≥ 2).

The results indicate that symptomatic knee OA worsens SF-6D health utility score significantly by 0.025 points (0.9 MID) on average. The association of unilateral and bilateral symptomatic knee OA with health utility were similar. Previously, it has been shown that symptomatic knee OA is associated with a decrease of 0.003–0.19 points in EQ-5D utility scores [17, 18]. However, the precise comparability of the results is challenging because of different preference-based health utility measures [32] and knee OA definitions utilized in previous studies: Muraki and co-authors [17] based their definition on the highest K-L grade of both knees, similarly to one of our definitions. They used K-L grade 3 as a cut-off point of radiographic knee OA, and symptomatic knee OA was defined as knee pain lasting at least 1 month within the current or previous year in the knee with K-L grade ≥ 3. Kiadaliri and co-authors [18] used clinical ACR (American College of Rheumatology) criteria based on frequent knee pain, crepitus, morning stiffness, age, and/or bony enlargements and a radiographic definition that approximates K-L grade 2 or higher. Both of the studies utilized unilateral knee OA definition (i.e., the OA status of the other knee was omitted).

We considered unilateral symptomatic and radiographic definitions (2-scale measures and the highest K-L grade) to be overly broad definition of health states in a sense of health economics analyses. That is why we wanted to examine, if we can differentiate the changes in disutility scores between bilateral definitions and the five (0–4) K-L grades. Because health utility is tied to a person’s general experience on quality of life but knee OA may emergence only in one or both knees, we used bilateral definitions of OA and the mean and combination of K-L grades as an attempt to take the effect of both knees on disutility into account, yet these are not validated and generally accepted outcome measures. Our results indicate that as symptomatic definition was applied, the unilateral and bilateral definitions yielded similar outcomes. On the other hand, according to our findings, we consider bilateral definition important when plain radiographic definition is applied.

Our findings indicate that the bilateral radiographic definition of knee OA and the mean and combination classifications of K-L grades were able to differentiate disutility scores between the health states although pain experience was not included in the OA definition. Mean K-L grade definition was able to differentiate more groups than the highest K-L grade. This may be result of the fact that K-L grades of both knees were included, even though rather heterogeneous K-L pairs result in the same mean value. The advantage of combination K-L grades is that it does neither omit nor summarize the K-L grades of the knees, but it resulted in smaller group sizes, which enlarged the reported confidence intervals.

The capability of K-L grade alone to differentiate the disutility between sequential health states was limited, although the difference between the extreme K-L grades was explicit in all definitions. For example, the definition based on the highest K-L grade was able to differentiate all pairwise disutility comparisons only with K-L grade 4. The reason for inadequate differentiation of disutility between the consecutive K-L grades may be that the radiographic classification has its shortcomings, and K-L is more likely an ordinal, not an interval scale. Firstly, the original K-L grades are verbally described, and the interpretation of them and radiographs is inconsistent and varies between observers [33]. Secondly, radiographic findings of knee OA does not absolutely result in pain experience or disability [34], which are the obvious factors deteriorating the patient-reported HRQoL. On the other hand, radiographic findings have some prognostic strength in relation to the symptomatology [35, 36].

Results of the present study confirm that the bilateral definition of knee OA was related to higher disutility scores if the definition was symptomatic, incorporating patient-reported frequent pain experience, rather than simple radiographic definition [17, 18]. Interestingly, our results are also consistent with previous findings that there is a relationship between self-reported knee pain and radiological OA defined by the highest K-L grade of both knees based on a previous study showing that knee pain experience differed by K-L grades and was explicit at least with the worst K-L grade [35].

Results of the present study may also be interpreted in the perspective of a health economic evaluation. If an intervention would prevent the progression of joint damage in both knees from the mean K-L grade 0 to 2 or from 2 to 4 the raw QALY gain would be 0.012, or 0.028 per year, respectively. In the treatment of OA, long-term objectives should focus on prevention of joint damage and on the improvement of quality of life [37].

If there was a more consistent diagnostic test for knee OA, defining the health states would be more straightforward. At present, the comparability of clinical and cost-effectiveness studies is challenging due to different definitions of knee OA. The comparability between studies is also challenging because of different applied health utility instruments. The present study applied the SF-12 questionnaire, and the results were converted to SF-6D-utilities by a mapping algorithm [20]. SF-6D takes into account six dimensions of the original SF-12 questionnaire. An advantage of the SF-6D measure is that it provides a single index score for the estimation of QALYs in cost-effectiveness analyses. We used the value of 0.027 [31] as a cut-point for MID as a smallest change in health utility that is important to a subject. However, estimates for SF-6D MID-values vary from 0.010 to 0.048 [38].

To our knowledge, the present study is the first to quantify the health disutility related to knee OA using the generic, preference-based SF-6D instrument in relation to different symptomatic and radiographic uni- and bilateral definitions of knee OA. Although the impact of knee OA on quality of life has been studied widely, there has been lack of information on preference-based quality of life in relation to different definitions of knee OA, and particularly in relation to uni- and bilateral definitions. Previously, it has been shown that bilateral knee pain is associated with poorer quality of life [10]. However, we found that bilateral knee OA definition is uncommon in HRQoL research, even though both knees naturally affect patients’ quality of life. This is probably a result of the fact that the generally used definitions of knee OA do not differentiate between uni- or bilateral knee OA. In the previous knee OA related quality of life, functional decline, and pain studies, the use of unilateral knee OA definition is usually implicitly expressed, and the selection of the analyzed knee has been variable (e.g., the worse knee or symptomatic definition with knee OA in at least one knee or knee randomly selected or both knees analyzed separately) (e.g., [17, 18, 25, 35, 39]). However, in our study we explicitly quantified the health disutility in relation to symptomatic and radiographic uni- and bilateral knee OA definitions.

The strength of this study is that we quantified the SF-6D scores in a repeated measures design with a substantial number of OAI study subjects. OAI is a large open access database with relatively low rate of drop-outs and high response rate to the validated SF-12 questionnaire. Earlier studies have quantified health utility in relation to knee OA using EQ-5D instrument in cross-sectional study design [17, 18]. There is no gold standard for generic preference-based HRQoL measure. National health technology assessment organizations such as NICE (National Institute for Health and Care Excellence) recommend EQ-5D as a generic health utility measure. However, a working group for standard set of outcome measures recently recommended both SF-12 and EQ-5D as standard patient reported outcome measure tools for persons with hip or knee OA for HRQoL evaluation [24]. Variety of disease-spesific instruments (KOOS, WOMAC, knee pain) may also be used to measure knee OA related quality of life. The OAI dataset includes KOOS, WOMAC and SF-12 questionnaires. Although the disease-spesific instruments have advantages, and they cover the dimensions relevant to knee OA, they are not suitable for calculating QALYs for health economic analyses unlike generic, preference-based health utility instruments. However, there are mapping algorithms available to estimate the preference-based utility from the disease-spesific instrument scores but they should be applied cautiously [40].

The results of the present study should be interpreted in light of some limitations. Firstly, while we have reported the health disutilities of OAI study participants, the findings of this study are, however, based on UK population-based preferences. There is evidence that health state valuing may differ between countries [41, 42]. SF-6D valuation surveys have been completed also in China [43], Japan [41], Portugal [44], Spain [45] and Brazil [46]. Secondly, as we took patient reported comorbid conditions into account we did not specifically focus on comorbidities of musculoskeletal system as adjusting variables. However, problems in musculoskeletal system, joint pain comorbidity, coexisting back pain, and hip OA have been reported to have impact on the quality of life and experience of knee pain in patients with knee OA [8, 47–50]. Thirdly, we used symptomatic definition only in 2- and 3-scale definitions. It would have been interesting to combine pain status also with the different radiographic definitions, but this would have resulted in too small group sizes. Fourthly, there was a substantial number of missing K-L grades at year 1, 2 and 3 follow-up visits. The GEE model utilized has a straightforward assumption of missing completely at random (MCAR). The missing K-L grades could have been ‘safely’ imputed with the technique of ‘last observation carried forward’ as the degeneration in knee joint (K-L grade) is not assumed to recover. However, 94 and 85% of participants had K-L grade available at baseline and at year 4 follow-up visit, respectively. Fifthly, the OAI study participants may be healthier and more educated than general population, which may limit the generalizability of the findings.

## Conclusions

The results of the present study confirm that knee OA is associated with diminished HRQoL. Our study expands upon the previous research by quantifying the health disutility values associated with different symptomatic and radiographic definitions of knee OA, as the gold standard of knee OA definition is currently unavailable. As previous research, we found different definitions of knee OA to be associated with different health disutilities, which can have an effect on the results of health economic analyses. Health disutility in relation to bilateral knee OA is less studied, and we found it to differ from disutility associated with unilateral knee OA when using radiographic definition of knee OA. The radiographic definition was, however, a crude measure to differentiate the disutility by sequential K-L grades. The performance of symptomatic definition was better, indicating that pain experience is an important factor in knee OA related quality of life.

## Additional files

Additional file 1 OAI datasets. This table reports specific OAI datasets, from where data for each subject were collected and, applied in analyses. (PDF 10 kb)

Additional file 2 Histograms of SF-6D score frequencies. These figures report distribution of SF-6D disutility scores presented as histograms and normal curves. (PDF 682 kb)

Additional file 3 Characteristics of participants and prevalence of knee OA. These tables report characteristics of participants eligible for data analyses and prevalence of knee OA according to different definitions during follow-up. (PDF 187 kb)

Additional file 4 SF-6D-disutility scores and parameter estimates. These tables reports estimated marginal means of SF-6D-disutility scores and 95% confidence intervals (CI) from GEE-analyses. (PDF 294 kb)

Additional file 5 Pairwise differences between SF-6D-disutility scores. These tables report pairwise differences between estimated marginal means of SF-6D-disutility scores from GEE-analyses. (PDF 151 kb)

## Abbreviations

ACR: American College of Rheumatology
BMI: Body mass index
EQ-5D: EuroQol-5 dimensions questionnaire
GEE: Generalized estimating equation model
HRQoL: Health-related quality of life
K-L: Kellgren-Lawrence radiographic system
KOOS: Knee injury and osteoarthritis outcome score
MCAR: Missing completely at random
MID: Minimally important difference
NICE: National Institute for Health and Care Excellence
OA: Osteoarthritis
OAI: Osteoarthritis Initiative
PASE: Physical activity scale for the elderly score
QALY: Quality-adjusted life year
SD: Standard deviation
SF-12: Short form health survey
WOMAC: Western Ontario and McMaster Universities osteoarthritis index
