# Supplementary material for: Health-related quality of life in relation to symptomatic and radiographic definitions of knee osteoarthritis: data from Osteoarthritis Initiative (OAI) 4-year follow-up study
Source: Health Qual Life Outcomes. 2018 Jul 31;16:154. doi: 10.1186/s12955-018-0979-7 (PMC6069966; doi:10.1186/s12955-018-0979-7)
Supplement: Supplementary file 1 — OAI datasets. This table reports specific OAI datasets, from where data for each subject were collected and, applied in analyses. (PDF 10 kb) [file 12955_2018_979_MOESM1_ESM.pdf]

Additional file 1 Health-Related Quality of Life in Relation to Symptomatic and Radiographic Definitions of Knee Osteoarthritis: Data from Osteoarthritis Initiative (OAI) 4-Year Follow-Up Study

This table reports specific OAI datasets, from where data for each subject were collected and, applied in analyses.

Table S1 OAI datasets analysed during the current study are available from the Osteoarthritis Initiative (OAI), http://www.oai.ucsf.edu/.

**OAI Dataset Label***
Enrollees_SAS
Outcomes99_SAS
AllClinical01_SAS
AllClinical03_SAS
AllClinical05_SAS
AllClinical06_SAS
kXR_SQ_BU00_SAS
kXR_SQ_BU01_SAS
kXR_SQ_BU03_SAS
kXR_SQ_BU05_SAS
SubjectChar06_SAS

*Data from baseline and follow-up visits at 12, 24, 36 and 48 months were applied in the study
