# Supplementary material for: Health-related quality of life in relation to symptomatic and radiographic definitions of knee osteoarthritis: data from Osteoarthritis Initiative (OAI) 4-year follow-up study
Source: Health Qual Life Outcomes. 2018 Jul 31;16:154. doi: 10.1186/s12955-018-0979-7 (PMC6069966; doi:10.1186/s12955-018-0979-7)

Additional file 2 Health-Related Quality of Life in Relation to Symptomatic and Radiographic Definitions of Knee Osteoarthritis: Data from Osteoarthritis Initiative (OAI) 4-Year Follow-Up Study

These figures report distribution of SF-6D disutility scores presented as histograms and normal curves.

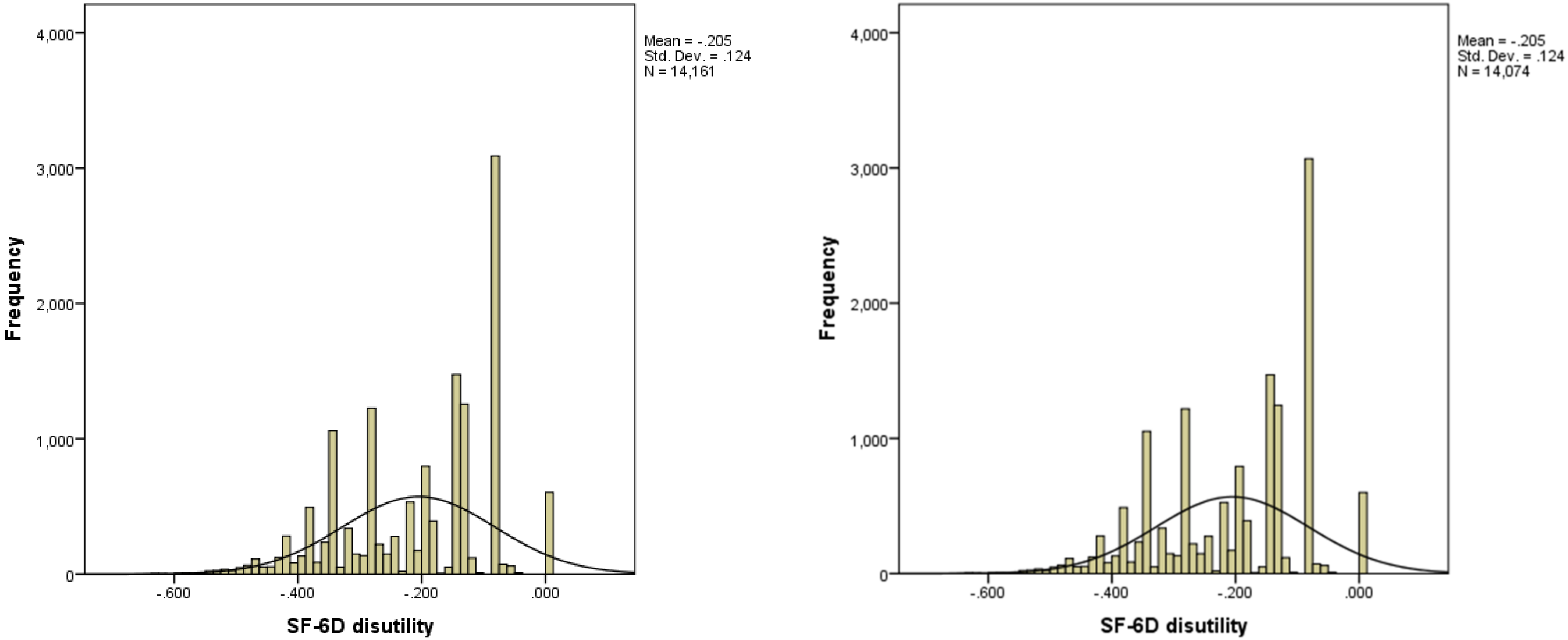

Figure S1. Histograms of SF-6D score frequencies and normal curves, radiographic (N=14,161) and symptomatic (N=14,074) definitions of knee OA

Additional file 2 Health-Related Quality of Life in Relation to Symptomatic and Radiographic Definitions of Knee Osteoarthritis: Data from Osteoarthritis Initiative (OAI) 4-Year Follow-Up Study

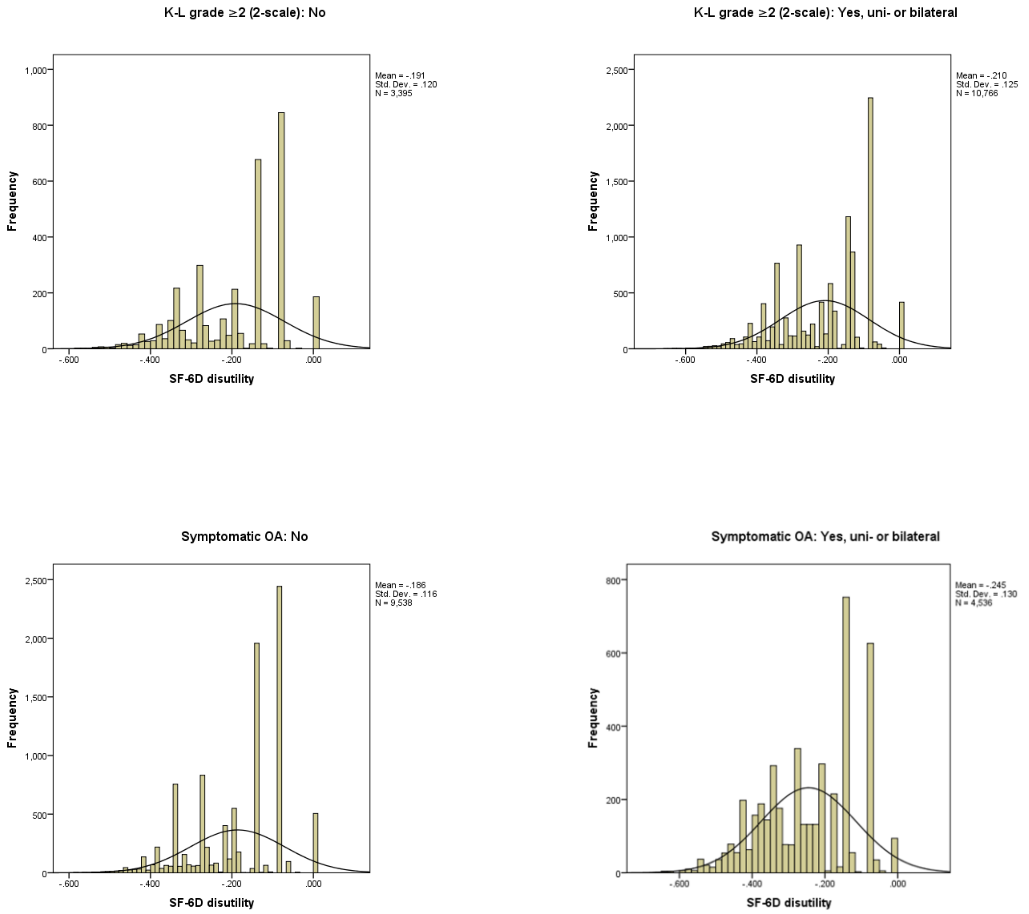

Figure S2. Histograms of SF-6D score frequencies and normal curves, radiographic (N=14,161) and symptomatic (N=14,074) definitions of knee OA

Additional file 2 Health-Related Quality of Life in Relation to Symptomatic and Radiographic Definitions of Knee Osteoarthritis: Data from Osteoarthritis Initiative (OAI) 4-Year Follow-Up Study

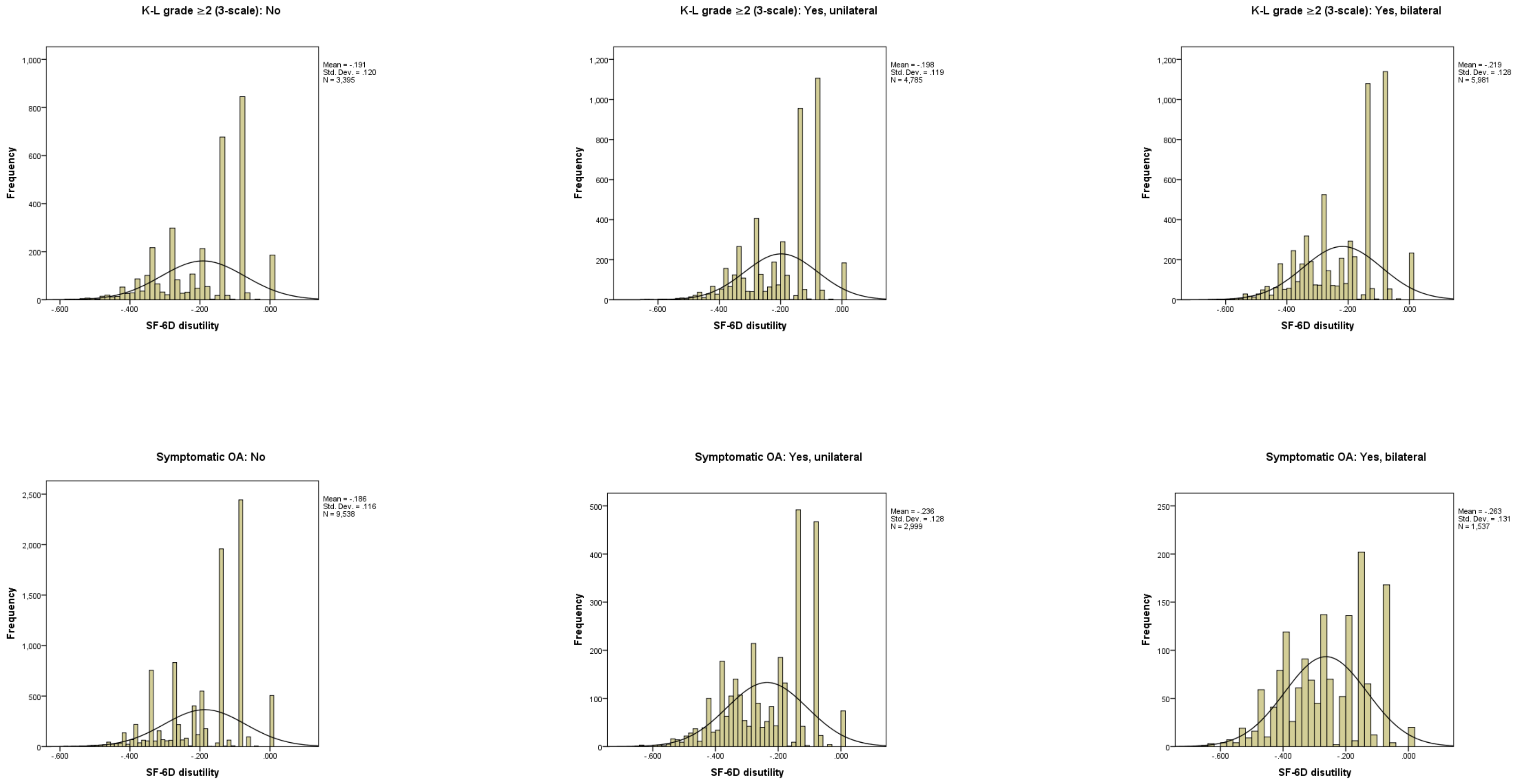

Figure S3. Histograms of SF-6D score frequencies and normal curves, radiographic (N=14,161) and symptomatic (N=14,074) definitions of knee OA

Additional file 2 Health-Related Quality of Life in Relation to Symptomatic and Radiographic Definitions of Knee Osteoarthritis: Data from Osteoarthritis Initiative (OAI) 4-Year Follow-Up Study

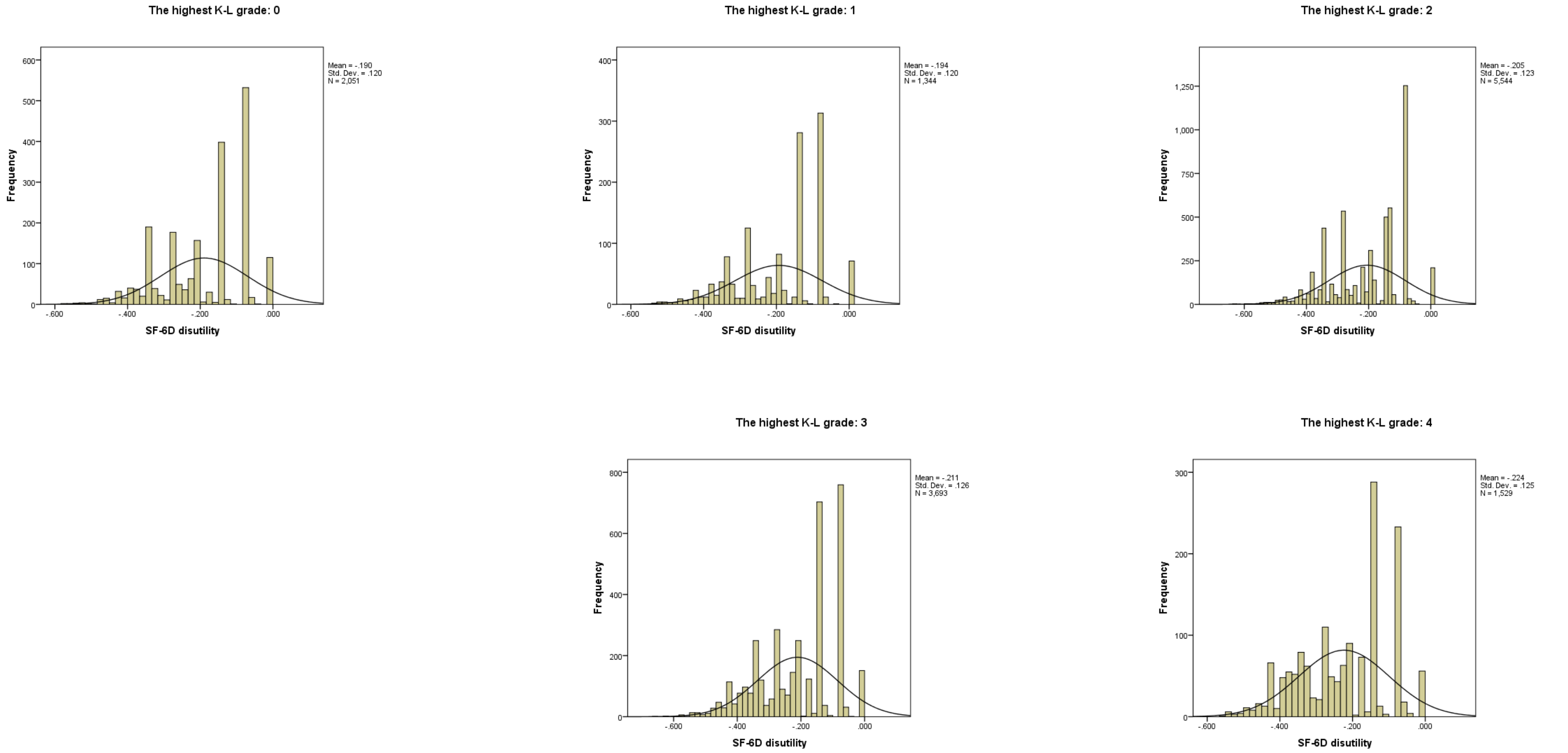

Figure S4. Histograms of SF-6D score frequencies and normal curves, radiographic definition of knee OA (N=14,161), the highest K-L grade

Additional file 2 Health-Related Quality of Life in Relation to Symptomatic and Radiographic Definitions of Knee Osteoarthritis: Data from Osteoarthritis Initiative (OAI) 4-Year Follow-Up Study

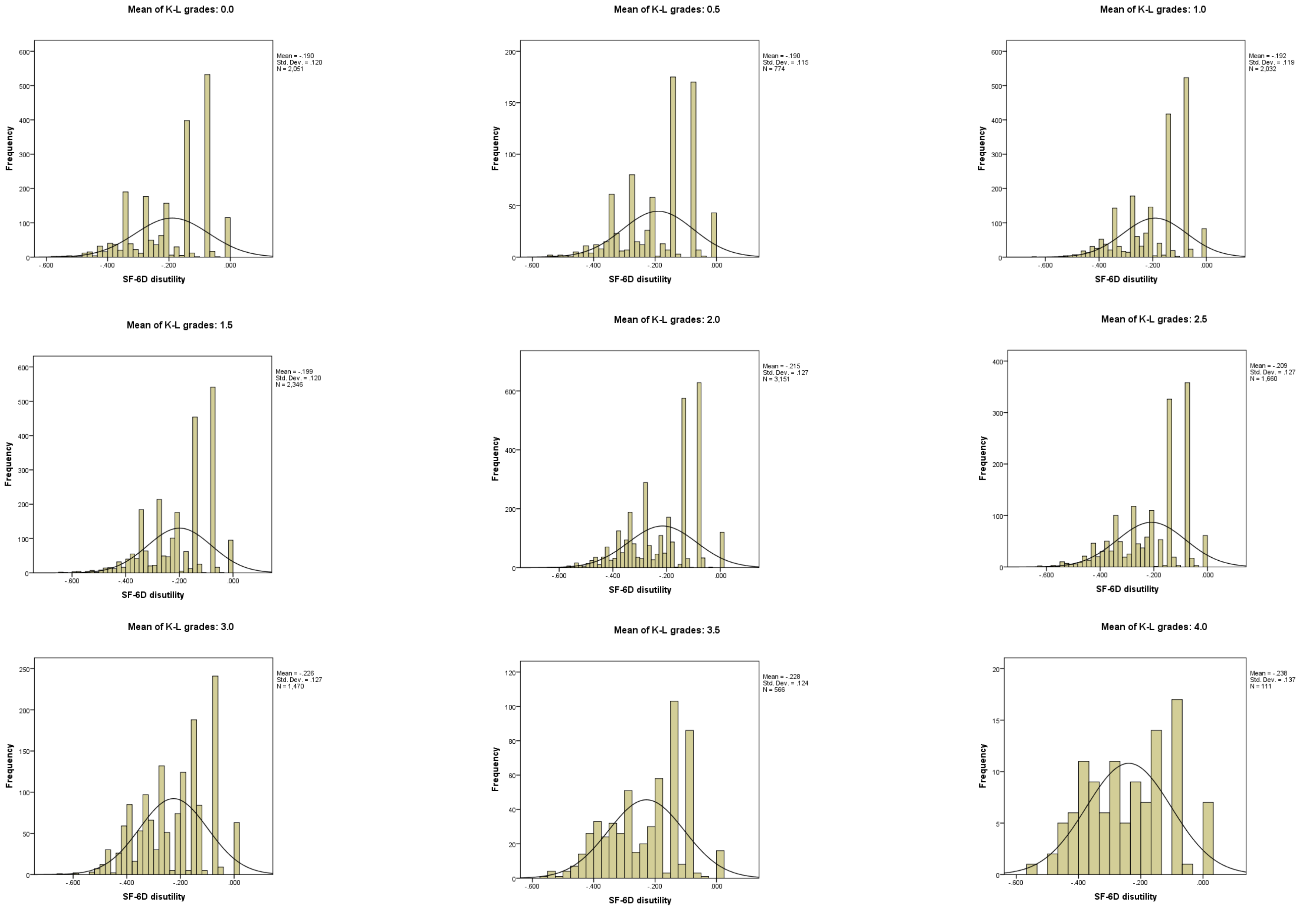

Figure S5. Histograms of SF-6D score frequencies and normal curves, radiographic definition of knee OA (N=14,161), mean of K-L grades

Additional file 2 Health-Related Quality of Life in Relation to Symptomatic and Radiographic Definitions of Knee Osteoarthritis: Data from Osteoarthritis Initiative (OAI) 4-Year Follow-Up Study

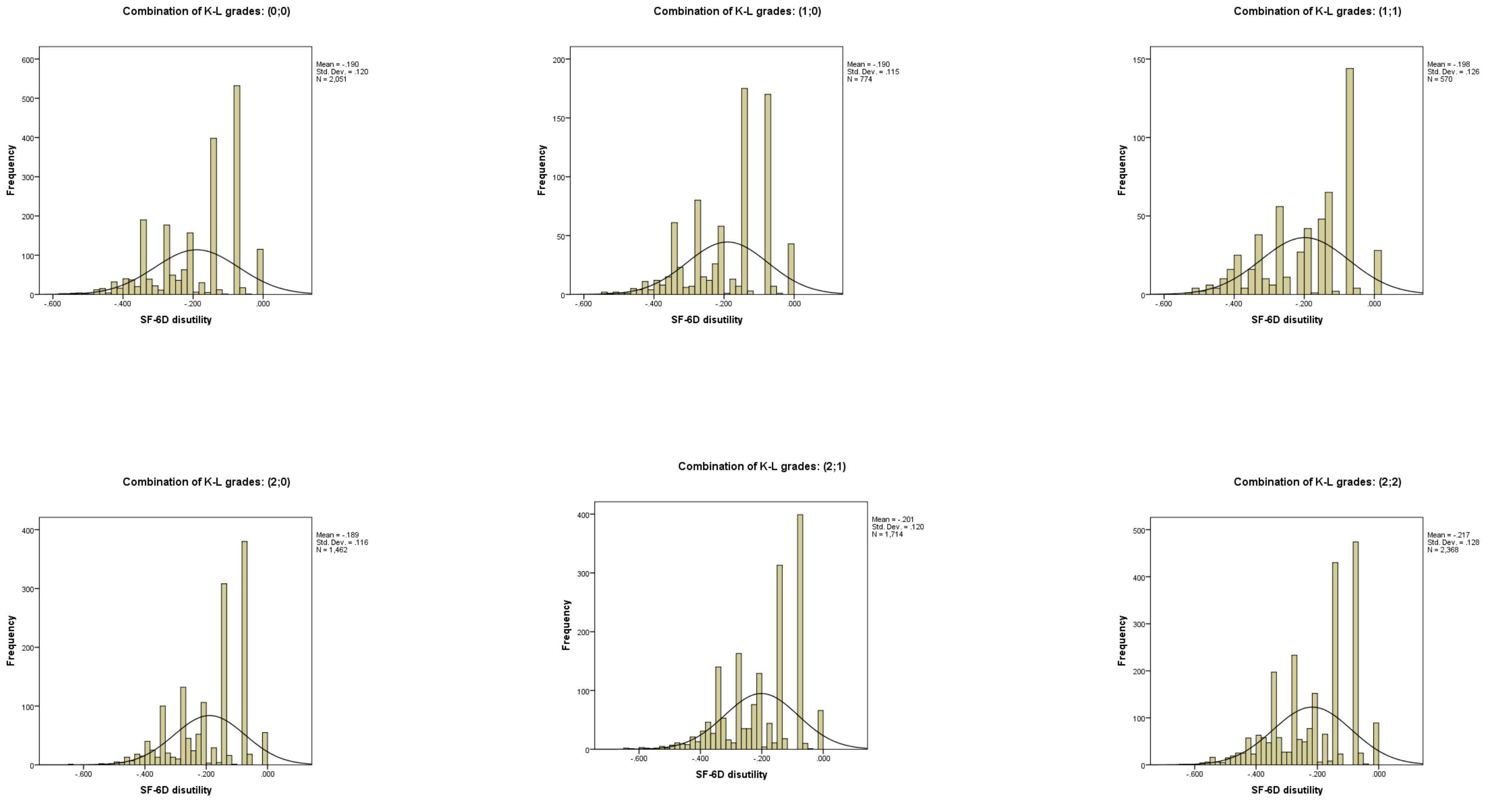

Figure S6. Histograms of SF-6D score frequencies and normal curves, radiographic definition of knee OA (N=14,161), combination of K-L grades (figure continues)

Additional file 2 Health-Related Quality of Life in Relation to Symptomatic and Radiographic Definitions of Knee Osteoarthritis: Data from Osteoarthritis Initiative (OAI) 4-Year Follow-Up Study

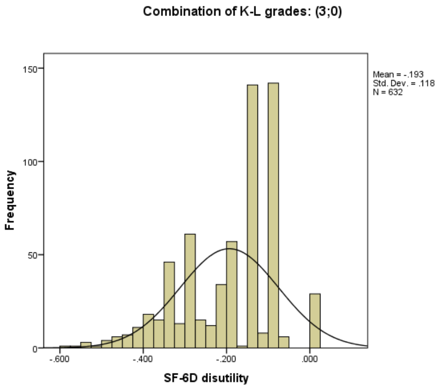

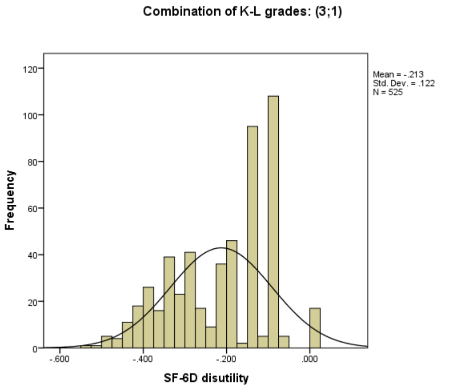

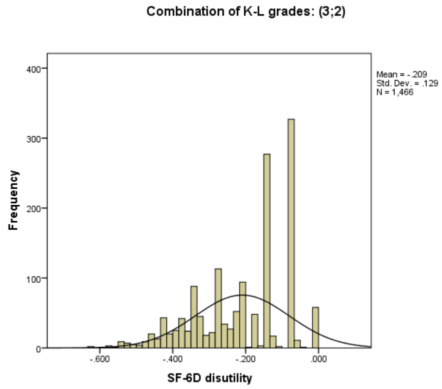

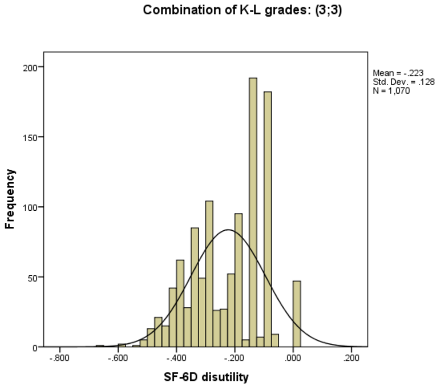

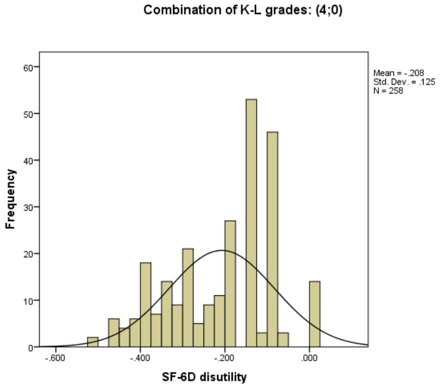

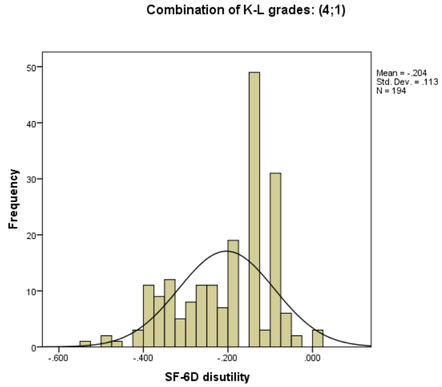

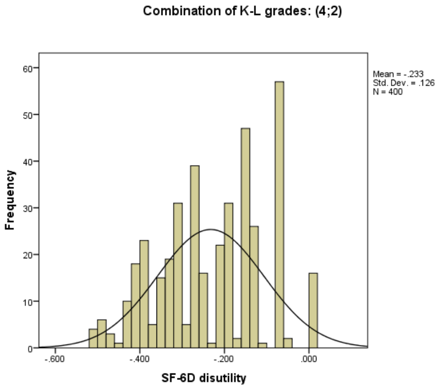

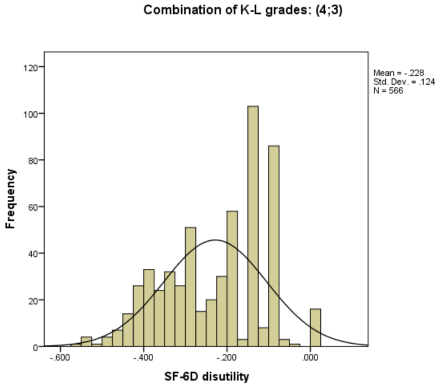

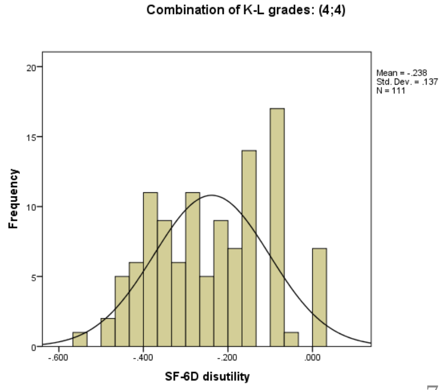

Supplement: Supplementary file 2 — Histograms of SF-6D score frequencies. These figures report distribution of SF-6D disutility scores presented as histograms and normal curves. (PDF 682 kb) [file 12955_2018_979_MOESM2_ESM.pdf]
