# Supplementary material for: Health-related quality of life in relation to symptomatic and radiographic definitions of knee osteoarthritis: data from Osteoarthritis Initiative (OAI) 4-year follow-up study
Source: Health Qual Life Outcomes. 2018 Jul 31;16:154. doi: 10.1186/s12955-018-0979-7 (PMC6069966; doi:10.1186/s12955-018-0979-7)
Supplement: Supplementary file 3 — Characteristics of participants and prevalence of knee OA. These tables report characteristics of participants eligible for data analyses and prevalence of knee OA according to different definitions during follow-up. (PDF 187 kb) [file 12955_2018_979_MOESM3_ESM.pdf]

These tables report number and characteristics of participants eligible for data analyses and prevalence of knee OA according to different definitions during follow-up.

Table S2 Characteristics of participants eligible for data analyses *

Variable	Baseline	Year 1	Year 2	Year 3	Year 4
**Participants**					
Participated study visit	4796	4496	4324	4270	4256
K-L grade available for both knees	4505 (93.9)	2596 (57.7)	2447 (56.6)	2354 (55.1)	3610 (84.8)
Eligible for analyses [1]	4278 (89.2)	2424 (53.9)	2249 (52.0)	2059 (48.2)	3151 (74.0)
Eligible for analyses [2]	4248 (88.6)	2409 (53.6)	2230 (51.6)	2053 (48.1)	3134 (73.6)
**Age, years**					
45–54	1264 (29.5)	491 (20.3)	371 (16.5)	294 (14.3)	461 (14.6)
55–64	1393 (32.6)	783 (32.3)	766 (34.1)	687 (33.4)	1138 (36.1)
>65	1621 (37.9)	1150 (47.4)	1112 (49.4)	1078 (52.4)	1552 (49.3)
**Gender**					
Male	1782 (41.7)	1020 (42.1)	951 (42.3)	868 (42.2)	1356 (43.0)
Female	2496 (58.3)	1404 (57.9)	1298 (57.7)	1191 (57.8)	1795 (57.0)
**Race**					
White or Caucasian	3458 (80.8)	1927 (79.5)	1803 (80.2)	1658 (80.5)	2616 (83.0)
Other	820 (19.2)	497 (20.5)	446 (19.8)	401 (19.5)	535 (17.0)
**Education †**					
Tertiary	1336 (31.2)	730 (30.1)	694 (30.9)	634 (30.8)	1011 (32.1)
Secondary	1277 (29.9)	713 (29.4)	663 (29.5)	632 (30.7)	1008 (32.0)
None / Primary	1665 (38.9)	981 (40.5)	892 (39.7)	793 (38.5)	1132 (35.9)
**Living status (number of persons) ‡**					
Live alone	928 (21.7)	549 (22.6)	499 (22.2)	490 (23.8)	714 (22.7)
Living with someone else	3350 (78.3)	1875 (77.4)	1750 (77.8)	1569 (76.2)	2437 (77.3)
**BMI, kg/m2**					
<25	1040 (24.3)	453 (18.7)	426 (18.9)	387 (18.8)	786 (24.9)
25 to <30	1690 (39.5)	950 (39.2)	871 (38.7)	849 (41.2)	1242 (39.4)
30 to <35	1122 (26.2)	703 (29.0)	670 (29.8)	576 (28.0)	788 (25.0)
≥35	426 (10.0)	318 (13.1)	282 (12.5)	247 (12.0)	335 (10.6)
**Comorbidities §**					
score 0	3254 (76.1)	1810 (74.7)	1603 (71.3)	1505 (73.1)	2234 (70.9)
score >0	1024 (23.9)	614 (25.3)	646 (28.7)	554 (26.9)	917 (29.1)
**Physical activity, PASE ¶**					
238–580	798 (18.7)	375 (15.5)	302 (13.4)	279 (13.6)	535 (17.0)
176–237	853 (19.9)	480 (19.8)	422 (18.8)	395 (19.2)	584 (18.5)
135–175	866 (20.2)	457 (18.9)	466 (20.7)	375 (18.2)	596 (18.9)
91–134	871 (20.4)	505 (20.8)	517 (23.0)	473 (23.0)	671 (21.3)
0–90	890 (20.8)	607 (25.0)	542 (24.1)	537 (26.1)	765 (24.3)
**Smoking status ‖**					
Never	1977 (46.2)	1116 (46.0)	1023 (45.5)	925 (44.9)	1587 (50.4)
Former	1908 (44.6)	1105 (45.6)	1034 (46.0)	956 (46.4)	1351 (42.9)
Current	393 (9.2)	203 (8.4)	192 (8.5)	178 (8.6)	213 (6.8)
**Knee injuries**					

No	2424 (56.7)	1196 (49.3)	1070 (47.6)	934 (45.4)	1593 (50.6)
Yes	1854 (43.3)	1228 (50.7)	1179 (52.4)	1125 (54.6)	1558 (49.4)
**Knee surgical history**					
No	3341 (78.1)	1697 (70.0)	1555 (69.1)	1398 (67.9)	2383 (75.6)
Yes	937 (21.9)	727 (30.0)	694 (30.9)	661 (32.1)	768 (24.4)
**Disutility (SF-6D) score, mean (SD)**	–0.199 (0.120)	–0.210 (0.125)	–0.208 (0.126)	–0.209 (0.124)	–0.205 (0.126)

Abbreviations: BMI Body Mass Index; Comorbidites Charlson comorbidity index score; K-L Kellgren-Lawrence radiographic system; PASE Physical Activity Scale for the Elderly; SD Standard Deviation

* Values are presented as N (%) unless stated otherwise

[1] Radiographic definition of knee OA

[2] Symptomatic definition of knee OA (pain questionnaire required)

† The highest grade of school completed: tertiary (graduate degree), secondary (college graduate or some graduate school), and primary/none level (less than college)

‡ OAI data covered living status at baseline and year 3 follow-up visit. Missing data at year 1, 2 and 4 was imputed with the information available from the baseline and year 3 visit.

§ OAI data covered comorbid conditions at baseline, year 2 and year 4 follow-up visits. Missing data at year 1 and 3 was imputed with the information available from the baseline and year 2 visit, respectively.

¶ Physical activity PASE score quintiles (higher scores indicate greater physical activity)

‖ OAI data covered smoking status at baseline and year 4 follow-up visit. Missing data at year 1, 2 and 3 was imputed with the information available from the baseline visit.

Table S3 Prevalence of knee OA according to different definitions during follow-up, N (%)

Variable	Baseline	Year 1	Year 2	Year 3	Year 4
**Participants**	4278	2424	2249	2059	3151
**Symptomatic OA status * (2-scale)**					
No	3134 (73.8)	1457 (60.5)	1338 (60.0)	1241 (60.4)	2368 (75.6)
Yes, uni- or bilateral	1114 (26.2)	952 (39.5)	892 (40.0)	812 (39.6)	766 (24.4)
Missing †	30	15	19	6	17
**Symptomatic OA status * (3-scale)**					
No	3134 (73.8)	1457 (60.5)	1338 (60.0)	1241 (60.4)	2368 (75.6)
Yes, unilateral	762 (17.9)	642 (26.7)	596 (26.7)	508 (24.7)	491 (15.7)
Yes, bilateral	352 (8.3)	310 (12.9)	296 (13.3)	304 (14.8)	275 (8.8)
Missing †	30	15	19	6	17
**K-L grade ≥2 (2-scale)**					
No	1860 (43.5)	120 (5.0)	84 (3.7)	39 (1.9)	1292 (41.0)
Yes, uni- or bilateral	2418 (56.5)	2304 (95.0)	2165 (96.3)	2020 (98.1)	1859 (59.0)
**K-L grade ≥2 (3-scale)**					
No	1860 (43.5)	120 (5.0)	84 (3.7)	39 (1.9)	1292 (41.0)
Yes, unilateral	1112 (26.0)	1019 (42.0)	953 (42.4)	884 (42.9)	817 (25.9)
Yes, bilateral	1306 (30.5)	1285 (53.0)	1212 (53.9)	1136 (55.2)	1042 (33.1)
**The highest K-L grade**					
0	1199 (28.0)	32 (1.3)	17 (0.8)	6 (0.3)	797 (25.3)
1	661 (15.5)	88 (3.6)	67 (3.0)	33 (1.6)	495 (15.7)
2	1288 (30.1)	1182 (48.8)	1103 (49.0)	1019 (49.5)	952 (30.2)
3	841 (19.7)	802 (33.1)	745 (33.1)	695 (33.8)	610 (19.4)
4	289 (6.8)	320 (13.2)	317 (14.1)	306 (14.9)	297 (9.4)
**Mean of K-L grades**					
0.0	1199 (28.0)	32 (1.3)	17 (0.8)	6 (0.3)	797 (25.3)
0.5	383 (9.0)	44 (1.8)	36 (1.6)	19 (0.9)	292 (9.3)
1.0	612 (14.3)	347 (14.3)	322 (14.3)	288 (14.0)	463 (14.7)
1.5	550 (12.9)	499 (20.6)	467 (20.8)	437 (21.2)	393 (12.5)
2.0	728 (17.0)	682 (28.1)	625 (27.8)	577 (28.0)	539 (17.1)
2.5	391 (9.1)	364 (15.0)	337 (15.0)	298 (14.5)	270 (8.6)
3.0	313 (7.3)	327 (13.5)	294 (13.1)	278 (13.5)	258 (8.2)
3.5	100 (2.3)	117 (4.8)	127 (5.6)	122 (5.9)	100 (3.2)
4.0	2 (0.0)	12 (0.5)	24 (1.1)	34 (1.7)	39 (1.2)
**Combination of K-L grades**					
(0;0)	1199 (28.0)	32 (1.3)	17 (0.8)	6 (0.3)	797 (25.3)
(1;0)	383 (9.0)	44 (1.8)	36 (1.6)	19 (0.9)	292 (9.3)
(1;1)	278 (6.5)	44 (1.8)	31 (1.4)	14 (0.7)	203 (6.4)
(2;0)	334 (7.8)	303 (12.5)	291 (12.9)	274 (13.3)	260 (8.3)
(2;1)	403 (9.4)	368 (15.2)	344 (15.3)	312 (15.2)	287 (9.1)
(2;2)	551 (12.9)	511 (21.1)	468 (20.8)	433 (21.0)	405 (12.9)
(3;0)	147 (3.4)	131 (5.4)	123 (5.5)	125 (6.1)	106 (3.4)
(3;1)	123 (2.9)	114 (4.7)	106 (4.7)	97 (4.7)	85 (2.7)
(3;2)	340 (7.9)	318 (13.1)	299 (13.3)	269 (13.1)	240 (7.6)
(3;3)	231 (5.4)	239 (9.9)	217 (9.6)	204 (9.9)	179 (5.7)

(4;0)	54 (1.3)	57 (2.4)	51 (2.3)	47 (2.3)	49 (1.6)
(4;1)	51 (1.2)	46 (1.9)	38 (1.7)	29 (1.4)	30 (1.0)
(4;2)	82 (1.9)	88 (3.6)	77 (3.4)	74 (3.6)	79 (2.5)
(4;3)	100 (2.3)	117 (4.8)	127 (5.6)	122 (5.9)	100 (3.2)
(4;4)	2 (0.0)	12 (0.5)	24 (1.1)	34 (1.7)	39 (1.2)

* K-L grade ≥2 and knee pain on more than half the days during past month in the same knee

† Pain questionnaire required for the definition of symptomatic knee OA

Table S4 Number of participants stratified according to number of observations available for analyses, N (%)

Observations, N	Symptomatic knee OA definition*	Radiographic knee OA definition
**5**	1504 (34.3)	1547 (35.3)
**4**	499 (11.4)	466 (10.6)
**3**	338 (7.7)	337 (7.7)
**2**	1501 (34.2)	1513 (34.5)
**1**	542 (12.4)	525 (12.0)
**In total**	**4384 (100.0)**	**4388 (100.0)**

* K-L grade ≥2 and knee pain on more than half the days during past month in the same knee (pain questionnaire required for the definition of symptomatic knee OA)
