# Supplementary material for: Health-related quality of life in relation to symptomatic and radiographic definitions of knee osteoarthritis: data from Osteoarthritis Initiative (OAI) 4-year follow-up study
Source: Health Qual Life Outcomes. 2018 Jul 31;16:154. doi: 10.1186/s12955-018-0979-7 (PMC6069966; doi:10.1186/s12955-018-0979-7)
Supplement: Supplementary file 4 — SF-6D-disutility scores and parameter estimates. These tables reports estimated marginal means of SF-6D-disutility scores and 95% confidence intervals (CI) from GEE-analyses. (PDF 294 kb) [file 12955_2018_979_MOESM4_ESM.pdf]

These tables report estimated marginal means of SF-6D-disutility scores, the parameter estimates, and 95% confidence intervals (CI) from GEE-analyses.

Table S5 Estimated marginal means of SF-6D-disutility scores and 95% confidence intervals (CI) from GEE-analyses

	Mean (95% CI)	p
**Symptomatic OA status † (2-scale)**		
No	–0.220 (–0.226;–0.215)	
Yes, uni- or bilateral	–0.246 (–0.252;–0.239)	<0.001*
**Symptomatic OA status † (3-scale)**		
No	–0.220 (–0.226;–0.214)	
Yes, unilateral	–0.244 (–0.250;–0.237)	<0.001*
Yes, bilateral	–0.250 (–0.258;–0.242)	<0.001*
**K-L grade ≥2 (2-scale)**		
No	–0.225 (–0.232;–0.218)	
Yes, uni- or bilateral	–0.231 (–0.237;–0.225)	0.040*
**K-L grade ≥2 (3-scale)**		
No	–0.224 (–0.231;–0.216)	
Yes, unilateral	–0.225 (–0.231;–0.218)	0.774
Yes, bilateral	–0.237 (–0.243;–0.230)	<0.001*
**The highest K-L grade**		
0	–0.223 (–0.231;–0.215)	
1	–0.225 (–0.233;–0.216)	0.706
2	–0.226 (–0.233;–0.220)	0.349
3	–0.231 (–0.238;–0.224)	0.055
4	–0.249 (–0.259;–0.240)	<0.001*
**Mean of K-L grades**		
0.0	–0.222 (–0.230;–0.213)	
0.5	–0.224 (–0.234;–0.214)	0.651
1.0	–0.218 (–0.226;–0.209)	0.375
1.5	–0.224 (–0.232;–0.216)	0.554
2.0	–0.234 (–0.242;–0.227)	0.004*
2.5	–0.230 (–0.239;–0.221)	0.118
3.0	–0.241 (–0.250;–0.231)	0.001*
3.5	–0.258 (–0.270;–0.246)	<0.001*
4.0	–0.262 (–0.284;–0.240)	0.001*
**Combination of K-L grades**		
(0;0)	–0.222 (–0.230;–0.213)	
(1;0)	–0.224 (–0.234;–0.214)	0.674
(1;1)	–0.221 (–0.234;–0.209)	0.969
(2;0)	–0.215 (–0.225;–0.206)	0.217
(2;1)	–0.225 (–0.234;–0.216)	0.476
(2;2)	–0.233 (–0.241;–0.225)	0.015*
(3;0)	–0.224 (–0.237;–0.211)	0.741
(3;1)	–0.231 (–0.244;–0.218)	0.196
(3;2)	–0.229 (–0.239;–0.220)	0.152
(3;3)	–0.240 (–0.250;–0.229)	0.003*
(4;0)	–0.250 (–0.271;–0.229)	0.010*

(4;1)	–0.232 (–0.256;–0.209)	0.375
(4;2)	–0.242 (–0.257;–0.227)	0.012*
(4;3)	–0.258 (–0.270;–0.246)	<0.001*
(4;4)	–0.262 (–0.284;–0.240)	0.001*

* The mean difference is significant at the $p<0.05$ level, p-values are presented in comparison to reference groups (“No OA” or “K-L grade 0”)

† K-L-grade ≥2 and knee pain on more than half the days during past month in the same knee

Table S6 Parameter estimates from GEE-analyses, symptomatic OA status (2-scale)

**Parameter**	**Category**	**B**	**95% CI**	**p**
**Symptomatic OA status † (2-scale)**	Yes, uni- or bilateral	–0.025	–0.029;–0.021	<0.001*
	No	1.0		
**Age, years**	>65	0.018	0.012;0.025	<0.001*
	55–64	0.007	0.001;0.013	0.022*
	45–54	1.0		
**Gender**	Female	–0.013	–0.019;–0.006	<0.001*
	Male	1.0		
**Race**	Other	–0.012	–0.021;–0.004	0.005*
	White or Caucasian	1.0		
**Education ‡**	None / Primary	–0.034	–0.041;–0.027	<0.001*
	Secondary	–0.011	–0.018;–0.004	0.002*
	Tertiary	1.0		
**Living status (number of persons)**	Living with someone else	0.015	0.009;0.022	<0.001*
	Live alone	1.0		
**BMI, kg/m2**	≥35	–0.031	–0.040;–0.022	<0.001*
	30 to <35	–0.015	–0.022;–0.008	<0.001*
	25 to <30	–0.003	–0.009;0.003	0.365
	<25	1.0		
**Comorbidities**	score >0	–0.023	–0.028;–0.018	<0.001*
	score 0	1.0		
**Physical activity, PASE §**	0–90	–0.026	–0.032;–0.020	<0.001*
	91–134	–0.013	–0.019;–0.008	<0.001*
	135–175	–0.004	–0.009;0.002	0.210
	176–237	–0.003	–0.008;0.002	0.292
	238–580	1.0		
**Smoking status**	Current	–0.030	–0.041;–0.020	<0.001*
	Former	–0.005	–0.011;0.001	0.074
	Never	1.0		
**Knee injuries**	Yes	–0.020	–0.026;–0.014	<0.001*
	No	1.0		
**Knee surgical history**	Yes	0.005	–0.002;0.012	0.169
	No	1.0		

*The difference is significant at the $p<0.05$ level

† K-L grade ≥2 and knee pain on more than half the days during past month in the same knee

‡ The highest grade of school completed: tertiary (graduate degree), secondary (college graduate or some graduate school), and primary/none level (less than college)

§ Physical activity PASE score quintiles (higher scores indicate greater physical activity)

Table S7 Parameter estimates from GEE-analyses, symptomatic OA status (3-scale)

**Parameter**		**B**	**95% CI**	**p**
**Symptomatic OA status † (3-scale)**	Yes, unilateral	–0.030	–0.036;–0.023	<0.001*
	Yes, bilateral	–0.024	–0.028;–0.019	<0.001*
	No	1.0		
**Age, years**	>65	0.018	0.012;0.025	<0.001*
	55–64	0.007	0.001;0.013	0.022*
	45–54	1.0		
**Gender**	Female	–0.012	–0.019;–0.006	<0.001*
	Male	1.0		
**Race**	Other	–0.012	–0.021;–0.004	0.006*
	White or Caucasian	1.0		
**Education ‡**	None / Primary	–0.034	–0.041;–0.027	<0.001*
	Secondary	–0.011	–0.018;–0.004	0.002*
	Tertiary	1.0		
**Living status (number of persons) ‡**	Living with someone else	0.015	0.009;0.022	<0.001*
	Live alone	1.0		
**BMI, kg/m2**	≥35	–0.030	–0.040;–0.021	<0.001*
	30 to <35	–0.015	–0.022;–0.008	<0.001*
	25 to <30	–0.003	–0.009;0.003	0.367
	<25	1.0		
**Comorbidities §**	score >0	–0.023	–0.028;–0.018	<0.001*
	score 0	1.0		
**Physical activity, PASE**	0–90	–0.026	–0.032;–0.020	<0.001*
	91–134	–0.013	–0.019;–0.008	<0.001*
	135–175	–0.004	–0.009;0.002	0.211
	176–237	–0.003	–0.008;0.002	0.297
	238–580	1.0		
**Smoking status**	Current	–0.030	–0.041;–0.020	<0.001*
	Former	–0.005	–0.011;0.000	0.073
	Never	1.0		
**Knee injuries**	Yes	–0.020	–0.026;–0.014	<0.001*
	No	1.0		
**Knee surgical history**	Yes	0.005	–0.002;0.012	0.164
	No	1.0		

*The difference is significant at the p<0.05 level

† K-L grade ≥2 and knee pain on more than half the days during past month in the same knee

‡ The highest grade of school completed: tertiary (graduate degree), secondary (college graduate or some graduate school), and primary/none level (less than college)

§ Physical activity PASE score quintiles (higher scores indicate greater physical activity)

Table S8 Parameter estimates from GEE-analyses, K-L grade ≥2 (2-scale)

**Parameter**	**Category**	**B**	**95% CI**	**p**
**K-L grade ≥2 (2-scale)**	Yes, uni- or bilateral	–0.006	–0.012;0.000	0.040*
	No	1.0		
**Age, years**	>65	0.017	0.011;0.024	<0.001*
	55–64	0.007	0.001;0.012	0.027*
	45–54	1.0		
**Gender**	Female	–0.013	–0.020;–0.007	<0.001*
	Male	1.0		
**Race**	Other	–0.015	–0.024;–0.006	0.001*
	White or Caucasian	1.0		
**Education ‡**	None / Primary	–0.035	–0.043;–0.028	<0.001*
	Secondary	–0.010	–0.018;–0.003	0.004*
	Tertiary	1.0		
**Living status (number of persons)**	Living with someone else	0.015	0.008;0.021	<0.001*
	Live alone	1.0		
**BMI, kg/m2**	≥35	–0.034	–0.044;–0.025	<0.001*
	30 to <35	–0.018	–0.025;–0.010	<0.001*
	25 to <30	–0.004	–0.010;0.002	0.215
	<25	1.0		
**Comorbidities**	score >0	–0.023	–0.028;–0.017	<0.001*
	score 0	1.0		
**Physical activity, PASE §**	0–90	–0.026	–0.032;–0.020	<0.001*
	91–134	–0.013	–0.019;–0.007	<0.001*
	135–175	–0.004	–0.009;0.002	0.206
	176–237	–0.003	–0.008;0.002	0.221
	238–580	1.0		
**Smoking status**	Current	–0.030	–0.041;–0.020	<0.001*
	Former	–0.005	–0.011;0.000	0.074
	Never	1.0		
**Knee injuries**	Yes	–0.020	–0.026;–0.014	<0.001*
	No	1.0		
**Knee surgical history**	Yes	0.002	–0.006;0.009	0.651
	No	1.0		

*The difference is significant at the p<0.05 level

† K-L grade ≥2 and knee pain on more than half the days during past month in the same knee

‡ The highest grade of school completed: tertiary (graduate degree), secondary (college graduate or some graduate school), and primary/none level (less than college)

§ Physical activity PASE score quintiles (higher scores indicate greater physical activity)

Table S9 Parameter estimates from GEE-analyses, K–L grade ≥2 (3-scale)

Parameter	Category	B	95% CI	p
**K-L grade ≥2 (3-scale)**	Yes, bilateral	–0.013	–0.020;–0.006	<0.001*
	Yes, unilateral	–0.001	–0.007;0.005	0.774
	No	1.0		
**Age, years**	>65	0.018	0.012;0.025	<0.001*
	55–64	0.007	0.001;0.013	0.019*
	45–54	1.0		
**Gender**	Female	–0.013	–0.019;–0.006	<0.001*
	Male	1.0		
**Race**	Other	–0.014	–0.023;–0.005	0.002*
	White or Caucasian	1.0		
**Education ‡**	None / Primary	–0.035	–0.042;–0.027	<0.001*
	Secondary	–0.010	–0.018;–0.003	0.004*
	Tertiary	1.0		
**Living status (number of persons)**	Living with someone else	0.015	0.008;0.021	<0.001*
	Live alone	1.0		
**BMI, kg/m2**	≥35	–0.033	–0.042;–0.023	<0.001*
	30 to <35	–0.017	–0.024;–0.010	<0.001*
	25 to <30	–0.004	–0.010;0.003	0.253
	<25	1.0		
**Comorbidities**	score >0	–0.023	–0.028;–0.017	<0.001*
	score 0	1.0		
**Physical activity, PASE §**	0–90	–0.026	–0.032;–0.020	<0.001*
	91–134	–0.013	–0.019;–0.007	<0.001*
	135–175	–0.004	–0.009;0.002	0.208
	176–237	–0.003	–0.008;0.002	0.212
	238–580	1.0		
**Smoking status**	Current	–0.030	–0.041;–0.020	<0.001*
	Former	–0.005	–0.011;0.000	0.067
	Never	1.0		
**Knee injuries**	Yes	–0.020	–0.026;–0.014	<0.001*
	No	1.0		
**Knee surgical history**	Yes	0.002	–0.005;0.009	0.600
	No	1.0		

*The difference is significant at the p<0.05 level

† K-L grade ≥2 and knee pain on more than half the days during past month in the same knee

‡ The highest grade of school completed: tertiary (graduate degree), secondary (college graduate or some graduate school), and primary/none level (less than college)

§ Physical activity PASE score quintiles (higher scores indicate greater physical activity)

Table S10 Parameter estimates from GEE-analyses, the highest K-L grade

Parameter	Category	B	95% CI	p
**The highest K-L grade**	4	–0.027	–0.037;–0.016	<0.001*
	3	–0.008	–0.017;0.000	0.055
	2	–0.004	–0.011;0.004	0.349
	1	–0.002	–0.011;0.007	0.706
	0	1.0		
**Age, years**	>65	0.019	0.012;0.026	<0.001*
	55–64	0.007	0.001;0.013	0.016*
	45–54	1.0		
**Gender**	Female	–0.014	–0.021;–0.007	<0.001*
	Male	1.0		
**Race**	Other	–0.015	–0.024;–0.007	0.001*
	White or Caucasian	1.0		
**Education ‡**	None / Primary	–0.035	–0.042;–0.028	<0.001*
	Secondary	–0.011	–0.018;–0.003	0.004*
	Tertiary	1.0		
**Living status (number of persons)**	Living with someone else	0.015	0.008;0.021	<0.001*
	Live alone	1.0		
**BMI, kg/m2**	≥35	–0.033	–0.043;–0.024	<0.001*
	30 to <35	–0.017	–0.024;–0.010	<0.001*
	25 to <30	–0.004	–0.010;0.003	0.248
	<25	1.0		
**Comorbidities**	score >0	–0.023	–0.028;–0.017	<0.001*
	score 0	1.0		
**Physical activity, PASE §**	0–90	–0.026	–0.032;–0.020	<0.001*
	91–134	–0.013	–0.018;–0.007	<0.001*
	135–175	–0.003	–0.009;0.002	0.232
	176–237	–0.003	–0.008;0.002	0.266
	238–580	1.0		
**Smoking status**	Current	–0.031	–0.042;–0.020	<0.001*
	Former	–0.005	–0.011;0.000	0.070
	Never	1.0		
**Knee injuries**	Yes	–0.019	–0.025;–0.013	<0.001*
	No	1.0		
**Knee surgical history**	Yes	0.004	–0.003;0.012	0.253
	No	1.0		

*The difference is significant at the p<0.05 level

† K-L grade ≥2 and knee pain on more than half the days during past month in the same knee

‡ The highest grade of school completed: tertiary (graduate degree), secondary (college graduate or some graduate school), and primary/none level (less than college)

§ Physical activity PASE score quintiles (higher scores indicate greater physical activity)

Table S11 Parameter estimates from GEE-analyses, mean of K-L grades

Parameter	Category	B	95% CI	p
**Mean of K-L grades**	4.0	–0.040	–0.063;–0.018	0.001*
	3.5	–0.036	–0.049;–0.023	<0.001*
	3.0	–0.019	–0.030;–0.008	0.001*
	2.5	–0.008	–0.018;0.002	0.118
	2.0	–0.012	–0.021;–0.004	0.004*
	1.5	–0.003	–0.011;0.006	0.554
	1.0	0.004	–0.005;0.013	0.375
	0.5	–0.002	–0.013;0.008	0.651
	0.0	1.0		
**Age, years**	>65	0.020	0.013;0.027	<0.001*
	55–64	0.008	0.002;0.014	0.011*
	45–54	1.0		
**Gender**	Female	–0.013	–0.020;–0.007	<0.001*
	Male	1.0		
**Race**	Other	–0.014	–0.022;–0.005	0.002*
	White or Caucasian	1.0		
**Education ‡**	None / Primary	–0.035	–0.042;–0.027	<0.001*
	Secondary	–0.010	–0.018;–0.003	0.005*
	Tertiary	1.0		
**Living status (number of persons)**	Living with someone else	0.015	0.008;0.021	<0.001*
	Live alone	1.0		
**BMI, kg/m2**	≥35	–0.032	–0.041;–0.022	<0.001*
	30 to <35	–0.016	–0.023;–0.009	<0.001*
	25 to <30	–0.003	–0.009;0.003	0.296
	<25	1.0		
**Comorbidities**	score >0	–0.023	–0.028;–0.017	<0.001*
	score 0	1.0		
**Physical activity, PASE §**	0–90	–0.026	–0.032;–0.020	<0.001*
	91–134	–0.013	–0.018;–0.007	<0.001*
	135–175	–0.004	–0.009;0.002	0.206
	176–237	–0.003	–0.008;0.002	0.250
	238–580	1.0		
**Smoking status**	Current	–0.031	–0.041;–0.020	<0.001*
	Former	–0.005	–0.011;0.000	0.058
	Never	1.0		
**Knee injuries**	Yes	–0.019	–0.025;–0.013	<0.001*
	No	1.0		
**Knee surgical history**	Yes	0.004	–0.004;0.012	0.295
	No	1.0		

*The difference is significant at the p<0.05 level

† K-L grade ≥2 and knee pain on more than half the days during past month in the same knee

‡ The highest grade of school completed: tertiary (graduate degree), secondary (college graduate or some graduate school), and primary/none level (less than college)

§ Physical activity PASE score quintiles (higher scores indicate greater physical activity)

Table S12 Parameter estimates from GEE-analyses, combination of K-L grades

Parameter	Category	B	95% CI	p
**Combination of K-L grades**	(4;4)	–0.040	–0.063;–0.017	0.001*
	(4;3)	–0.036	–0.049;–0.023	<0.001*
	(4;2)	–0.021	–0.036;–0.005	0.012*
	(4;1)	–0.011	–0.035;0.013	0.375
	(4;0)	–0.028	–0.050;–0.007	0.010*
	(3;3)	–0.018	–0.030;–0.006	0.003*
	(3;2)	–0.008	–0.018;0.003	0.152
	(3;1)	–0.009	–0.023;0.005	0.196
	(3;0)	–0.002	–0.016;0.011	0.741
	(2;2)	–0.011	–0.021;–0.002	0.015*
	(2;1)	–0.003	–0.013;0.006	0.476
	(2;0)	0.006	–0.004;0.016	0.217
	(1;1)	0.000	–0.012;0.013	0.969
	(1;0)	–0.002	–0.012;0.008	0.674
	(0;0)	1.0		
**Age, years**	>65	0.020	0.013;0.027	<0.001*
	55–64	0.008	0.002;0.014	0.011*
	45–54	1.0		
**Gender**	Female	–0.013	–0.020;–0.007	<0.001*
	Male	1.0		
**Race**	Other	–0.014	–0.023;–0.005	0.002*
	White or Caucasian	1.0		
**Education ‡**	None / Primary	–0.035	–0.042;–0.027	<0.001*
	Secondary	–0.010	–0.018;–0.003	0.004*
	Tertiary	1.0		
**Living status (number of persons)**	Living with someone else	0.015	0.008;0.021	<0.001*
	Live alone	1.0		
**BMI, kg/m2**	≥35	–0.032	–0.041;–0.023	<0.001*
	30 to <35	–0.016	–0.024;–0.009	<0.001*
	25 to <30	–0.003	–0.010;0.003	0.286
	<25	1.0		
**Comorbidities**	score >0	–0.023	–0.028;–0.017	<0.001*
	score 0	1.0		
**Physical activity, PASE §**	0–90	–0.026	–0.032;–0.019	<0.001*
	91–134	–0.013	–0.018;–0.007	<0.001*
	135–175	–0.003	–0.009;0.002	0.220
	176–237	–0.003	–0.008;0.002	0.264
	238–580	1.0		
**Smoking status**	Current	–0.031	–0.041;–0.020	<0.001*
	Former	–0.005	–0.011;0.000	0.063
	Never	1.0		
**Knee injuries**	Yes	–0.019	–0.025;–0.013	<0.001*
	No	1.0		
**Knee surgical history**	Yes	0.004	–0.003;0.012	0.272

	No	1.0

*The difference is significant at the $p<0.05$ level

† K-L grade ≥2 and knee pain on more than half the days during past month in the same knee

‡ The highest grade of school completed: tertiary (graduate degree), secondary (college graduate or some graduate school), and primary/none level (less than college)

§ Physical activity PASE score quintiles (higher scores indicate greater physical activity)
