# Supplementary material for: Health-related quality of life in relation to symptomatic and radiographic definitions of knee osteoarthritis: data from Osteoarthritis Initiative (OAI) 4-year follow-up study
Source: Health Qual Life Outcomes. 2018 Jul 31;16:154. doi: 10.1186/s12955-018-0979-7 (PMC6069966; doi:10.1186/s12955-018-0979-7)
Supplement: Supplementary file 5 — Pairwise differences between SF-6D-disutility scores. These tables report pairwise differences between estimated marginal means of SF-6D-disutility scores from GEE-analyses. (PDF 151 kb) [file 12955_2018_979_MOESM5_ESM.pdf]

These tables report pairwise differences between estimated marginal means of SF-6D-disutility scores from GEE-analyses.

Table S13 Pairwise differences between estimated marginal means of SF-6D-disutility score from GEE-analyses, symptomatic OA status (2-scale), K-L grade ≥2 and knee pain on more than half the days during past month in the same knee

Symptomatic OA status	No	Yes
**No**		–0.025*
**Yes**	0.025*	

*The mean difference is significant at the p<0.05 level

Table S14 Pairwise differences between estimated marginal means of SF-6D-disutility score from GEE-analyses, K-L grade ≥2 (2-scale)

K-L grade ≥2	No	Yes
**No**		–0.006*
**Yes**	0.006*	

*The mean difference is significant at the p<0.05 level

Table S15 Pairwise differences between estimated marginal means of SF-6D-disutility score from GEE-analyses, symptomatic OA status (3-scale), K-L grade ≥2 and knee pain on more than half the days during past month in the same knee

Symptomatic OA status	No	Yes, unilateral	Yes, bilateral
**No**		–0.024*	–0.030*
**Yes, unilateral**	0.024*		–0.006
**Yes, bilateral**	0.030*	0.006	

*The mean difference is significant at the p<0.05 level

Table S16 Pairwise differences between estimated marginal means of SF-6D-disutility score from GEE-analyses, K-L grade ≥2 (3-scale)

K-L grade ≥2	No	Yes, unilateral	Yes, bilateral
**No**		–0.001	–0.013*
**Yes, unilateral**	0.001		–0.012*
**Yes, bilateral**	0.013*	0.012*	

*The mean difference is significant at the p<0.05 level

Table S17 Pairwise differences between estimated marginal means of SF-6D-disutility score from GEE-analyses, the highest K-L grade

K-L grade	0	1	2	3	4
**0**		–0.002	–0.004	–0.008	–0.027*
**1**	0.002		–0.002	–0.007	–0.025*
**2**	0.004	0.002		–0.005	–0.023*
**3**	0.008	0.007	0.005		–0.018*
**4**	0.027*	0.025*	0.023*	0.018*	

*The mean difference is significant at the p<0.05 level

Table S18 Pairwise differences between estimated marginal means of SF-6D-disutility score from GEE-analyses, mean of K-L grades

K-L grade	0.0	0.5	1.0	1.5	2.0	2.5	3.0	3.5	4.0
**0.0**		–0.002	0.004	–0.003	–0.012*	–0.008	–0.019*	–0.036*	–0.040*
**0.5**	0.002		0.006	0.000	–0.010	–0.006	–0.017*	–0.034*	–0.038*
**1.0**	–0.004	–0.006		–0.007	–0.016*	–0.012*	–0.023*	–0.040*	–0.044*
**1.5**	0.003	0.000	0.007		–0.010*	–0.005	–0.016*	–0.034*	–0.038*
**2.0**	0.012*	0.010	0.016*	0.010*		0.005	–0.006	–0.024*	–0.028*
**2.5**	0.008	0.006	0.012*	0.005	–0.005		–0.011*	–0.028*	–0.032*
**3.0**	0.019*	0.017*	0.023*	0.016*	0.006	0.011*		–0.017*	–0.021
**3.5**	0.036*	0.034*	0.040*	0.034*	0.024*	0.028*	0.017*		–0.004
**4.0**	0.040*	0.038*	0.044*	0.038*	0.028*	0.032*	0.021	0.004	

*The mean difference is significant at the p<0.05 level

Table S19 Pairwise differences between estimated marginal means of SF-6D-disutility score from GEE-analyses, combination of K-L grades

**K-L grades**	**(0;0)**	**(1;0)**	**(1;1)**	**(2;0)**	**(2;1)**	**(2;2)**	**(3;0)**	**(3;1)**	**(3;2)**	**(3;3)**	**(4;0)**	**(4;1)**	**(4;2)**	**(4;3)**	**(4;4)**
**(0;0)**		–0.002	0.000	0.006	–0.003	–0.012*	–0.002	–0.009	–0.008	–0.018*	–0.028*	–0.011	–0.021*	–0.036*	–0.040*
**(1;0)**	0.002		0.002	0.008	–0.001	–0.009	0.000	–0.007	–0.005	–0.016*	–0.026*	–0.009	–0.018*	–0.034*	–0.038*
**(1;1)**	0.000	–0.002		0.006	–0.004	–0.012	–0.003	–0.009	–0.008	–0.018*	–0.029*	–0.011	–0.021*	–0.036*	–0.040*
**(2;0)**	–0.006	–0.008	–0.006		–0.010	–0.018*	–0.008	–0.015*	–0.014*	–0.024*	–0.034*	–0.017	–0.027*	–0.042*	–0.046*
**(2;1)**	0.003	0.001	0.004	0.010		–0.008	0.001	–0.006	–0.004	–0.015*	–0.025*	–0.007	–0.017*	–0.033*	–0.037*
**(2;2)**	0.012*	0.009	0.012	0.018*	0.008		0.009	0.002	0.004	–0.007	–0.017	0.001	–0.009	–0.025*	–0.029*
**(3;0)**	0.002	0.000	0.003	0.008	–0.001	–0.009		–0.007	–0.005	–0.016*	–0.026*	–0.008	–0.018	–0.034*	–0.038*
**(3;1)**	0.009	0.007	0.009	0.015*	0.006	–0.002	0.007		0.002	–0.009	–0.019	–0.002	–0.011	–0.027*	–0.031*
**(3;2)**	0.008	0.005	0.008	0.014*	0.004	–0.004	0.005	–0.002		–0.011	–0.021	–0.003	–0.013	–0.029*	–0.033*
**(3;3)**	0.018*	0.016*	0.018*	0.024*	0.015*	0.007	0.016*	0.009	0.011		–0.010	0.007	–0.002	–0.018*	–0.022*
**(4;0)**	0.028*	0.026*	0.029*	0.034*	0.025*	0.017	0.026*	0.019	0.021	0.010		0.018	0.008	–0.008	–0.012
**(4;1)**	0.011	0.009	0.011	0.017	0.007	–0.001	0.008	0.002	0.003	–0.007	–0.018		–0.010	–0.025	–0.029
**(4;2)**	0.021*	0.018*	0.021*	0.027*	0.017*	0.009	0.018	0.011	0.013	0.002	–0.008	0.010		–0.016	–0.020
**(4;3)**	0.036*	0.034*	0.036*	0.042*	0.033*	0.025*	0.034*	0.027*	0.029*	0.018*	0.008	0.025	0.016		–0.004
**(4;4)**	0.040*	0.038*	0.040*	0.046*	0.037*	0.029*	0.038*	0.031*	0.033*	0.022*	0.012	0.029	0.020	0.004	

*The mean difference is significant at the p<0.05 level
